# Animal Food Products to Support Human Nutrition and to Boost Human Health: The Potential of Feedstuffs Resources and Their Metabolites as Health-Promoters

**DOI:** 10.3390/metabo14090496

**Published:** 2024-09-13

**Authors:** Mario Cuchillo-Hilario, Mareli-Itzel Fournier-Ramírez, Margarita Díaz Martínez, Sara Montaño Benavides, María-Concepción Calvo-Carrillo, Silvia Carrillo Domínguez, María-Elena Carranco-Jáuregui, Elizabeth Hernández-Rodríguez, Patricia Mora-Pérez, Yesica R. Cruz-Martínez, Claudia Delgadillo-Puga

**Affiliations:** 1 Departamento de Nutrición Animal Dr. Fernando Pérez-Gil Romo, Instituto Nacional de Ciencias Médicas y Nutrición Salvador Zubirán (INCMNSZ), Ciudad de México 14080, Mexico; margarita.diazm@incmnsz.mx (M.D.M.); sara.montanob@incmnsz.mx (S.M.B.); concepcion.calvoc@incmnsz.mx (M.-C.C.-C.); silvia.carrillod@incmnsz.mx (S.C.D.); elena.carrancoj@incmnsz.mx (M.-E.C.-J.); yekann123@gmail.com (Y.R.C.-M.); 2 Departamento de Ciencias Biológicas, Facultad de Estudios Superiores de Cuautitlán, Universidad Nacional Autónoma de México, Km 3.5 Carretera Teoloyucan-Cuautitlán, Estado de México 54000, Mexico; marelyfournier@gmail.com

**Keywords:** phytochemical, bioactive compounds, secondary metabolism, antinutritional factors, phenols, antioxidant activity

## Abstract

Recent attention has been given to animal feeding and its impact on human nutrition. Animal feeding is essential for meeting human dietary needs, making it a subject of significant interest and investigation. This review seeks to outline the current understanding of this disciplinary area, with a focus on key research areas and their potential implications. The initial part of the paper discusses the importance of animal feed resources and recognizes their crucial role in guaranteeing sufficient nutrition for both humans and animals. Furthermore, we analyzed the categorization of animal feeds based on the guidelines established by the National Research Council. This approach offers a valuable structure for comprehending and classifying diverse types of animal feed. Through an examination of this classification, we gain an understanding of the composition and nutritional content of various feedstuffs. We discuss the major categories of metabolites found in animal feed and their impact on animal nutrition, as well as their potential health advantages for humans. Flavonoids, polyphenols, tannins, terpenoids, vitamins, antioxidants, alkaloids, and essential oils are the primary focus of the examination. Moreover, we analyzed their possible transference into animal products, and later we observed their occurrence in foods from animal sources. Finally, we discuss their potential to promote human health. This review offers an understanding of the connections among the major metabolites found in feedstuffs, their occurrence in animal products, and their possible impact on the health of both animals and humans.

## 1. Introduction

Over the years, interest has dramatically risen in exploring the possibility of using animal food products to enhance human nutrition as well as human health. Animal-derived food products are proven to be good sources of vital nutrients such as proteins, vitamins, minerals, oils, and bioactive compounds, essential for optimal health [1]. Although the interest of animal-derived foods in human nutrition goes beyond conventional feed resources, it reached the exploration of the possibility of harnessing the potential of nonconventional resources and their metabolites and how livestock might be able to feed on them [2,3]. It is necessary to examine deeper into the occurrence of metabolites in animal feedstuffs, how they are transformed into animal products by the animal, and how the animal metabolites can be harnessed in the support of human nutrition as well as enhancing human health. This paper will discuss these stated issues by presenting the major classes of metabolites occurring in feedstuffs and their significance in animal nutrition, as well as the possible health benefits to humans from these animal metabolites. In conclusion, this paper will highlight the importance of introducing animal-derived metabolites in a balanced diet.

Analysis of animal feedstuffs has identified several bioactive compounds such as peptides, lipids, essential oils, saponins, alkaloids, vitamins, and antioxidants [4,5,6,7]. These metabolites are involved in a wide variety of functions that are relevant to animal feeding through stimulating growth, boosting immune function, regulating gut health, and/or influencing specific metabolic pathways. Recent studies have concentrated on unraveling the mechanism of action of the metabolites and their bioavailability and bioactivity [4,5,6]. Consequently, feedstuffs and their metabolites powerfully control animal metabolism, such activity changes under conditions of obesity, metabolite existence in feedstuffs, potential quantity of such metabolite level, along with intake. For instance, studies have demonstrated that bioactive peptides derived from animal proteins can exhibit antihypertensive, antimicrobial, and antioxidant activities [8]. Such findings highlight the potential of animal-derived metabolites as functional ingredients with significant implications for human health [1,9,10]. Animal-based food products have long been acknowledged as sources for humans [1,11,12]. Meat, dairy items, eggs, and fish offer top notch proteins, vitamins, minerals, and lipids, essential compounds for growth, development, and overall health [11,13,14]. These nutrients play a role during life stages such as childhood, pregnancy, and breastfeeding. Moreover, nutrients from animal sources have been linked to enhanced functions, increased muscle mass and strength, and reduced the risk of conditions such as heart disease and osteoporosis [9,15]. Despite the possible threats involved, animal-based food products offer a wide range of bioactive compounds that can positively impact human health. Bioactive substances sourced from animals, such as carotenoids, polyunsaturated fatty acids, and peptides, have been linked to health advantages such as anti-inflammatory, immune regulating, and anticancer properties [16,17,18]. These bioactive compounds play a role in preventing and managing illnesses such as cancer, heart diseases, and neurodegenerative conditions [2,19]. Additionally, metabolites derived from animals have displayed effects in influencing the balance of gut bacteria, which is crucial for human wellbeing and disease prevention. The interaction between animal metabolites and gut bacteria can impact processes such as nutrient absorption and immune system function with multiple health benefits [11,20].

Inclusion of metabolites derived from animals in a balanced diet is crucial for ensuring optimal nutrition and health [9,21]. While plant-based eating plans have become popular, it is important to recognize that animal-based foods offer unique nutrients that may be difficult to solely obtain from plants. Adding animal-derived products and their bioactive components to one’s diet can help fulfill nutrient needs and improve the bioavailability and absorption of certain nutrients [6,11,20]. Additionally, the combined effects of different nutrients and phytonutrients found in animal products can contribute to their overall animal and their effect on human health benefits [12,14,22]. Therefore, understanding the key types of metabolites in feedstuffs and their impact on animal nutrition is essential to understand how they can enhance both animal food products and human health. By including animal-derived metabolites in a balanced diet, individuals can optimize their nutrient intake and take advantage of the health benefits these resources offer. Thus, incorporating animal food items into a balanced diet is highly recommended, especially when considering the specific nutritional needs of various populations and age groups. This study aims to investigate how metabolites present in animal feedstuffs can support human nutrition and promote human health.

In the present review, a descriptive-exhaustive bibliographic search of scientific papers published from 2000 to 2024 was conducted in different repositories: PubMed, Scopus, Cochrane, Repositorio UNAM, Google Scholar, Science Direct, Web of Science, SciELO and Redalyc. The keywords used were metabolites, bioactive compounds, feedstuffs, animal nutrition, poultry, health, meat, milk, cheese, egg, fish, animal by-products, phytochemicals, biotransformation, bioavailability, employing AND, OR, and NOT as major booleans.

This paper will discuss these stated issues by presenting the major classes of metabolites occurring in feedstuffs and their significance in animal nutrition, as well as the possible health benefits to humans from these animal metabolites. In conclusion, this paper will highlight the importance of introducing animal-derived metabolites in a balanced diet.

## 2. Animal Feedstuffs Classification

The significance of animal-based food items in upholding nutrition and promoting wellbeing, should not be underestimated. Like humans, animals also need care and a varied, balanced diet. The National Research Council has developed a system for classifying animal feeds and organizing sources according to their nutritional content [23]. The NRCs classification system for animal feed offers a guide for comprehending and enhancing livestock nutrition. The NRC includes eight feed classes, namely, class 1: dry forages; class 2: pasture, range plants, and forages fed fresh; class 3: silages; class 4: energetic sources; class 5: protein sources; class 6: minerals; class 7: vitamins; and class 8: additives (Table 1).

### 2.1. NRC Feed Class 1 (Dry Forages and Roughages) and NRC Feed Class 2 (Pasture, Range Plants, and Forages Fed Fresh; Both with ≥18% of Fiber)

Roughage plays a role in the diets of livestock, offering a source of fiber. This includes pasture and hay, any grass primarily. Research has emphasized the significance of roughage in enhancing rumen health and reducing the chances of metabolic issues in animals [34]. High quality roughage supports activity in the rumen, leading to improved fiber digestion and nutrient absorption. The interaction between feed components and their phytochemicals and metabolites, along with compounds and phytochemical content, is crucial for animal nutrition [3]. Phytochemicals are natural plant compounds that offer effects on animal wellbeing. These substances can function as antioxidants, antimicrobials, and anti-inflammatory agents. May even possess anticancer properties. The presence and levels of phytochemicals in feed ingredients in roughage or bulky forages and their fiber content can significantly influence animal health and performance [5,20].

Roughage and bulky forages, for example, hay and pasture, are sources of phytochemicals because they have a high fiber content. Researchers are also exploring sources such as algae, which offer potential as nutrient- and energy-rich feed [35]. Fiber plays a role in carrying phytochemicals, allowing for their release and extended exposure to the animal’s digestive system. This extended exposure improves the absorption and utilization of these compounds, leading to animal health. Moreover, feed rich in fiber can promote the growth of gut bacteria, further aiding in the metabolism of phytochemicals and enhancing animal wellbeing [1,36]. It has been observed that the phytochemical levels in feed can vary based on factors such as plant type, maturity stage, growing conditions, and processing methods used. For instance, drying and storing forages can reduce content due to degradation processes. Therefore, it is essential for nutritionists and producers to take these factors into account when designing diets to ensure animals receive adequate fiber intake. Researchers have conducted studies to identify phytochemicals in different feed types and assess their impact on animal health and performance [13,20,33,37,38]. These research projects have given us information about how plant-based feed with phytochemicals can help to improve the health of animals and to decrease the reliance on synthetic additives.

### 2.2. NRC Feed Class 3 (Silages)

Silages are fermented feeds that are primarily made from green forage crops such as maize, sorghum, and grasses. While this is common in temperate climate regions in tropical countries, it may also include forages such as grasses, shrubs, roots, agricultural leftovers, tree foliage, and other mixtures [39,40]. Researchers have extensively explored silage production methods and additives to enhance fermentation processes and preserve the value of silages while improving their stability [41,42,43]. Silages usually have high moisture levels while they are undergoing lactic fermentation that alters the chemical composition of the feed. Throughout fermentation, microorganisms such as lactic acid bacteria convert carbohydrates into acids such as lactic, propionic, and acetic acids. These acids play a role in preserving the feed by creating an environment that hampers the growth of harmful microorganisms. These processes of fermentation are essential to maintaining protein integrity and optimizing energy availability for animal growth and animal productivity [44].

The way silage is fermented can impact the types of compounds produced in it and some bioactive properties are also preserved. Various studies have looked into the metabolites and active substances found in types of silage. For instance, research indicates that sorghum grain can contain a range of bioactive phytochemicals, such as carotenoids, phenolic compounds, and flavonoids, known for their properties and potential health advantages [45]. When sorghum grain is used to make silage, the fermentation process may alter the levels of these compounds [46]. On the one hand, grass silage might contain isoflavones, which have been researched for their antioxidant effects [47]. The content of these compounds in silages can differ based on factors such as the type of plants used, their maturity when harvested, how they are ensiled, and their storage conditions. These variables play a role in determining the presence and amounts of plant-based chemicals and active substances in silages [43]. Therefore, it is crucial for animal nutritionists to take into account the qualities of the silage while designing animal diets to ensure consumption of these beneficial compounds.

### 2.3. NRC Feed Class 4 (Energetic Sources)

Energetic sources such as grains, oilseeds, and food industry byproducts offer an energy source for animal diets. These ingredients usually contain carbohydrates, fats, and oils for meeting the energy needs of livestock. Recent studies have been looking into ways to improve the use of these feedstuffs as energy sources through mainly processing methods and/or feed formulations [48]. Also, grains such as corn, wheat, and barley are well known for their abundance of phytochemicals and bioactive compounds such as flavonoids and carotenoids. These compounds have interesting bioactive properties, e.g., free radical scavengers, that contribute to the well-being of animals. Research indicates that they can positively impact animal health by reducing stress and inflammation while boosting some biological function such as immunity. Oilseeds such as soybeans, canola, and sunflower seeds also serve as sources of phytochemicals [49].

These ingredients have elements such as phytosterols, tocopherols, and polyphenols. The first are known to help lower cholesterol levels in animals and support heart health. The second, a type of vitamin E, acts as an antioxidant that shields cells from harm caused by oxidation. Polyphenols exhibit properties that may help combat inflammation and prevent cancer development. The presence of phytochemicals and bioactive compounds in energy sources can vary based on factors such as the type of plant used, how it is processed, and storage conditions. Procedures, in particular milling, heat treatment, and extraction methods, can impact whether these compounds are preserved or break down. Thus, it is crucial to consider how processing affects the content when creating diets from energetic sources. These ingredients have elements such as phytosterols, tocopherols, and polyphenols [50].

### 2.4. NRC Feed Class 5 (Protein Sources)

Livestock rely on a variety of protein sources to meet their amino acid needs, with traditional options such as soybean, canola, and fishmeal being commonly used. However, due to growing concerns about sustainability and the environment, researchers are now looking into unconventional protein sources [51]. Insect-based proteins, single-cell proteins, and products from fermentation have emerged as alternatives [52]. These innovative protein sources offer a chance to reduce reliance on feeds while still maintaining animal health and performance. Rich in amino acids, legumes, oilseeds, and animal by-products also play a role in providing amino acids to animals [53]. Additionally, these protein sources contain phytochemicals and bioactive compounds that support animal wellbeing. Legumes such as soybeans, peas, and lentils stand out as protein sources with phytochemical profiles that include isoflavones, saponins, and phenolic compounds. The first has specifically been associated with health benefits, such as inflammatory and antioxidant properties [16,17,18]. Saponins have demonstrated some antimicrobial and immunomodulatory effects. Also, phenolic compounds bring antioxidant benefits, which may contribute to the health of animals [11]. Oilseeds are mainly seen as energy sources. However, once their oils are extracted, the residual material is utilized to supplement animal diets [54]. These feed ingredients contain lignans, phytosterols, and tocopherols. Lignans show promise in preventing cancer and supporting heart health. Phytosterols help lower cholesterol levels, while tocopherols act as antioxidants that shield cells from damage caused by oxidation. Animal-derived products such as fish meal and meat meal can also serve as protein sources and bioactive compounds [9,20,55]. These feeds provide omega-3 fatty acids, peptides, and essential trace elements. Omega-3 fatty acids offer inflammatory properties and cardiovascular advantages. Peptides from animal proteins exhibit effects and immune system regulation capabilities [53]. The diversity of phytochemicals and bioactive compounds in protein sources can vary based on factors such as the type of plants or animals used and processing techniques employed [2]. Methods such as heat treatment or extraction processes can impact the preservation or breakdown of these compounds significantly. Therefore, it is crucial to consider how processing methods affect the content when designing animal diets.

### 2.5. NRC Feed Class 6 (Minerals) and NRC Feed Class 7 (Vitamins)

Minerals and vitamins play a role in the diets of livestock, aiding in metabolic functions, growth, and reproduction. Recent research has emphasized the importance of meeting mineral and vitamin needs in animal nutrition and refining methods for supplementing them to boost performance and immune response. Therefore, selenium represents a critical trace mineral pivotal in maintaining homeostasis in animals and humans. Substantial research indicates that supplementing the diet with selenium positively affects overall animal health, primarily attributable to its immunomodulatory properties and capacity to protect against oxidative damage. Moreover, it exhibits potential antiviral activity by safeguarding immune cells from oxidative damage and reducing viral replication and antioxidant activity [29]. Vitamin D deficiency in humans is a significant health concern. It is important to explore more effective food-based strategies to increase vitamin D intake. One promising approach is biofortifying animal feeds to produce sustainable, vitamin D-enriched foods; for example, pork meat, as estimated by Neill et al. [56] in four theoretical scenarios, suggesting that this method has the potential to fulfill up to 25% of individuals’ estimated average requirement of vitamin D. Furthermore, researchers have explored the benefits of additives such as prebiotics, probiotics, and enzymes in enhancing absorption, gut health, and the general welfare of animals.

### 2.6. NRC Feed Class 8 (Additives)

Some ingredients have been discovered to have an impact on animal feed in many ways, such as probiotics, prebiotics, organic acids, and antibiotics, among others [32,57]. For instance, probiotics are living microorganisms that offer health benefits to animals when they are consumed in adequate amounts. Some studies indicate that probiotics can improve the absorption and utilization of phytochemicals encountered in feedstuffs. In contrast, prebiotics are non-digestible ingredients that stimulate the growth and activity of beneficial gut bacteria. Therefore, some phytochemicals are either prebiotics or probiotics and can modulate gut microbiome [58]. Other components, such as organic acids, such as formic acid and propionic acid, are commonly used to preserve animal feed. These acids can change the acidity levels in the system, influencing how plant chemicals are digested and utilized [59]. Another category of additives with effects on plant chemical content is antioxidants. Antioxidants shield cells from harm caused by radicals. They can be natural or synthetic. When added to animal feed, they can impact the stability and bioavailability of phytochemicals. For instance, vitamin E, a recognized antioxidant, has been proven to enhance the stability of carotenoids [60].

## 3. Biochemistry of Major Classes of Natural Compounds Found in Animal Feedstuffs

A natural product is a chemical compound from living organisms such as bacteria, fungi, lichens, plants, and animals that can be isolated and thus characterized. The origin of these compounds is secondary metabolism, so they are also known as secondary metabolites. A major function of these compounds is to interact with molecular targets or receptors to facilitate the host’s metabolism response [2]. These natural products are classified into four major groups: terpenes, phenolic compounds, glycosides, and alkaloids.

### 3.1. Terpenes

**Figure 1:**
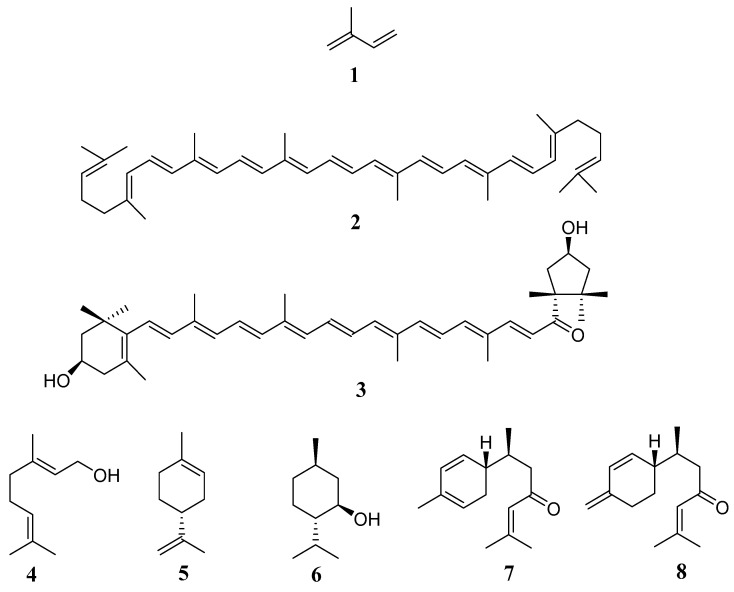
Terpenes present in natural products.

The largest family of natural products are the terpenes (Figure 1), whose biosynthesis occurs in the mevalonic acid pathway and in the methylerythritol phosphate pathway [2]. They are composed of isoprenoids, which are isoprene units with five carbon atoms (**1**). The number of isoprene units in their structure is what allows them to be classified as monoterpenes (C10), sesquiterpenes (C15), diterpenes (C20), triterpenes (C30), etc. These metabolites participate in various functions such as growth regulation, the formation of photosynthetic pigments such as phytol, carotenoids such as lycopene (**2**) or capsanthin (**3**), as well as being part of the structure of compounds such as phytosterols. The attraction they exert on some pollinators and seed dispersers is considered another of their functions. The volatile compounds responsible for the aroma of flowers and plants are complex mixtures, mainly of mono and sesquiterpenes, which result in essential oils, such as geraniol (**4**), (+)-limonene (**5**), (−)-menthol (**6**), *α*-turmerone (**7**), and *β*-turmerone (**8**). They can also contribute to water retention and antibacterial activity in plants [61,62].

### 3.2. Phenolic Compounds

**Figure 2:**
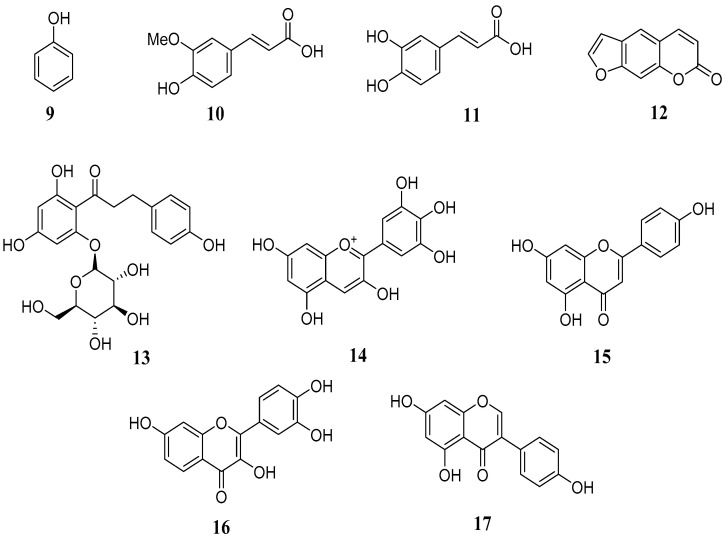
Phenolic compounds present in natural products.

Living organisms can synthesize a variety of secondary metabolites with a phenol group (**9**) in their structure, which are known as phenolic compounds as a result of the metabolism of aromatic amino acids such as phenylalanine, tryptophan, and tyrosine. This synthesis may involve the shikimic acid pathway and, less frequently, the malonic acid pathway [15,18]. Phenolic compounds (Figure 2) can also be analyzed from a structural perspective, which includes compounds such as: (**a**) ferulic acid (**10**) or caffeic acid (**11**) (simple molecules), (**b**) psoralen (**12**) (a coumarin), (**c**) phlorizin (a chalcones) (**13**), (**d**) flavonoids, which, according to their degree of oxidation, can be classified into anthocyanidins such as delphinidin (**14**), flavones such as apigenin (**15**), flavanols such as fisetin (**16**), and isoflavones such as genistein (**17**), and (**e**) polymers such as tannins and lignins [4,10]. These compounds are present in the metabolism of plants when reproduction and growth processes take place; they also perform a protection role against some pathogens and act as a defense when ultraviolet radiation occurs. They are present in some flowers, vegetables, and fruits and are responsible for their pigmentation as well as some sensory characteristics such as astringency. Their main potential benefit is their antioxidant activity and those properties that can be related to anti-inflammatory, antibacterial, and anticancer possible effects [16,17,18]. The intestinal epithelial barrier separates animal internal circulation from the intestinal environment and prevents pathogenic bacterial invasion. A variety of phenolic compounds, including some of those mentioned above, have been reported to improve intestinal epithelial barrier function and reduce and/or repair intestinal damage by regulating intestinal tight junction protein expression and paracellular permeability [11,16].

### 3.3. Glycosides

**Figure 3:**
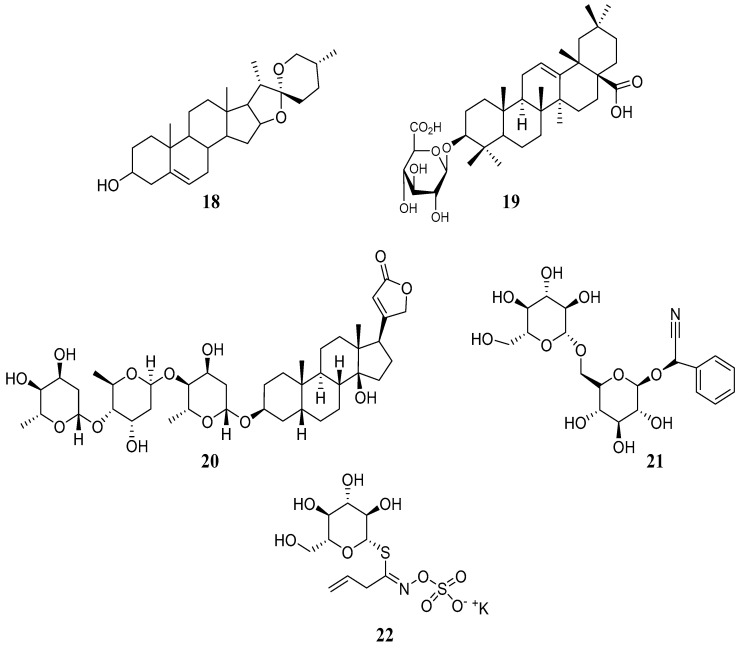
Glycosides present in natural products.

These compounds are secondary metabolites formed when a sugar molecule (*glycone*) condenses with another molecule (*aglycone*), which contains a hydroxyl group. Glycosides (Figure 3) include saponins, such as diosgenin (steroidal glycoside) (**18**) or calenduloside E (triterpenoid glycoside) (**19**); cardiac glycosides, such as digitoxin (**20**); cyanogenic glycosides such as amygdalin (**21**); and glucosinolates such as sinigrin (**22**) [63]. Glycosides participate in such important functions in plants, including defense against herbivores and pathogens, pigmentation of flowers and fruits, which results in attracting pollinators, growth regulation, and cell formation. They also affect the sensory profile of foods. Their medicinal potential has been recognized for treating heart diseases and studied for their anti-inflammatory and antimicrobial properties [64,65].

### 3.4. Alkaloids

**Figure 4:**
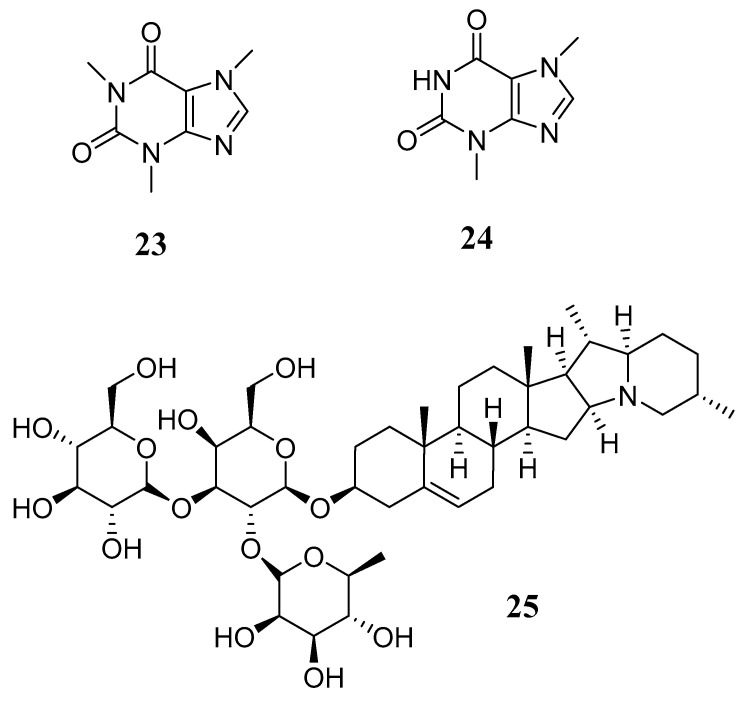
Alkaloids present in natural products.

These secondary metabolites are produced as a response to adaptation, immunity, and regulation processes. They are characterized by the presence of at least one nitrogen atom in their structure, which becomes protonated at a pH of 7.2 and causes them to behave as basic compounds in solution. They are produced from the metabolism of amino acids such as lysine, tyrosine, tryptophan, or ornithine. These compounds can be categorized into pseudoalkaloids and true alkaloids since they have or do not lack heterocyclic rings (amines and amides), which feature distinct ring structures. The most studied alkaloids include caffeine (**23**), theobromine (**24**) and solanine (**25**) (Figure 4), and it should be noted that alkaloids can be toxic in high doses due to their interaction with neurotransmitters. However, when administered in low doses, they act as muscle relaxants, tranquilizers, cough suppressants, or analgesics, which is why they represent an option when looking for therapeutic options [66,67].

There are so many different researchers trying to identify not only new functional and bioactive compounds but their mechanism of action. In this section, some of the most representative metabolites are mentioned due to their potential properties that could facilitate the prevention of chronic diseases such as obesity, diabetes, and cardiovascular disease, as well as improve well-being among healthy individuals. Considering the potential effects of other ingredients of livestock feed, such as vitamins or fatty acids, which are already well documented, the consumption of these compounds could result in a reduction of healthcare expenses.

## 4. Food Products of Animal Origin: Metabolites and Health Benefits and Implications

### 4.1. Milk and Dairy By-Products

Advances in animal production have highlighted the benefits of sustainable farming systems for human nutrition, wellbeing, and health. Studies have identified various bioactive compounds in milk and milk byproducts (Table 2), including polyunsaturated fatty acids, proteins (caseins, albumins), flavonoids, catechins, hydroxycinnamic acids, immunoglobulins (IgG, IgM, IgE, IgD), cytokines (interleukins, interferon), phenols, peptides, and terpenes, and essential oils, among others [8,9,10,68]. These metabolites, which are transferred through animal feeding, significantly impact the quality of animal-derived products [12,18]. Farming practices, such as grazing, tend to increase the proportion of linoleic and linolenic acids, leading to milk with better sensory qualities and a longer shelf life. Understanding the metabolic pathways of these compounds and their effects on human health is crucial for maximizing their beneficial impact. Practical recommendations based on this knowledge can guide dietary choices to enhance human health outcomes. Research has shown that specific plant species, such as Acacia, possess unique properties beyond their nutritional value, including anti-inflammatory and antioxidant effects, potentially preventing obesity and related disorders [69,70,71]. Uushona and colleagues [13] investigated the phytochemical properties of *Acacia mearnsii* and *A. dealbata* species for potential use in livestock nutrition. The authors noted that leaves collected during the hot- dry season have higher levels of crude protein, ether extract, and essential amino acids compared to those collected in the cool-wet season. This variation based on seasons is crucial for farmers and livestock breeders as it indicates that harvesting, at times, can optimize the advantages of these leaves for ruminants. Both species contain polyphenols and flavonoids, bioactive compounds with antioxidant properties. The antioxidants in these leaves can help reduce stress in ruminants, potentially leading to growth rates, improved feed conversion efficiency, and stronger immune responses. The research emphasizes that incorporating Acacia leaf meals into diets can significantly enhance animal production metrics, making them a valuable component of livestock feeding strategies. By using plants such as *A. mearnsii* and *A. dealbata*, farmers can tackle the issues of animal feed availability and expensive feed prices. These plants, typically viewed as invasive species, have the potential to be turned into feed options supporting friendly farming methods. According to the authors, incorporating these Acacia leaves into the diets of animals can decrease dependence on feed supplies, leading to a boost in biodiversity and ecological harmony within agricultural settings. Therefore, supplementing animal diets with bioactive compounds-rich plants can enhance the bioactivity of dairy products, supporting antioxidant activity and further modulating chronic diseases [7,72,73]. However, there remains a gap regarding the traceability of these compounds from ingested plants to dairy products and their specific health benefits on animals and humans.

Simitzis and Deligeorgis [6] highlight the significance of utilizing by-products from agriculture in animal nutrition to enhance the quality of animal products and promote wellbeing. The study highlights the importance of incorporating antioxidants in animal diets to improve oxidative stability and safeguard consumer health. The most important metabolites reported by those authors include lycopene (tomato pomace), hesperidin, naringin (citrus pulp), tyrosol (olive cake), betalains (sugar-beet pulp), punicalagins (pomegranate pulp), resveratrol (grape pomace), and quercetin (apple). The study also emphasizes the need for a comprehension of how animals metabolize these antioxidants to tailor supplementation strategies based on factors such as age, health status, and productivity. For example, grape pomace in sheep diet supplementation at 75% inclusion had negative effects on pH, ammonia, cellulolytic, and proteolytic activity in the rumen environment. Conversely, pomegranate peel (450 g/kg) reduces protein digestibility, ruminal ammonia, and volatile fatty acid concentrations in sheep and dairy cows [6]. These results indicate that integration of by-products and natural antioxidants into animal diets can enhance animal performance, elevate meat quality, and potentially offer health advantages for consumers [11]. Given these insights, further exploration into the biochemical mechanisms of these compounds is necessary. Understanding how animals absorb, metabolize, and utilize plant metabolites is essential, for optimizing their incorporation into diets. This understanding can help shape the creation of methods for animal supplementing, which could result in the generation of superior animal products and enhanced wellbeing for both animals and people.

Zhang and colleagues [55] explored the impact of consuming milk on health, highlighting its advantages. This study indicated that milk intake can enhance human health rather than cause detrimental effects. The research emphasizes the importance of milk in maintaining a healthy diet, supported by evidence analysis. For example, a daily increase of 200 mL in milk consumption diminishes the risk of heart disease, stroke, high blood pressure, colorectal cancer, metabolic issues, obesity, diabetes, Alzheimer’s disease, and bone-related problems. These results are highly significant as they demonstrate how milk could help address health concerns. Nevertheless, there are some cautions to consider, as the study linked milk intake with prostate cancer, Parkinson’s disease, skin issues such as acne, and iron deficiency in infants. In the same way, some of the benefits exhibited are related to bioactive metabolites found in milk, since proteins can be digested and generate the bioactive peptides, which are connected with a decreasing hypertension risk. Also, they pointed out that milk-derived tripeptides presented BP-lowering effects and that the emerging functional ingredient “milk polar lipids”, which is a nature component of the milk fat globule membrane, can significantly reduce the lipid biomarkers for CVD, including TC/HDL-C and apolipoprotein (Apo)B/ApoA1 ratios, by reducing intestinal cholesterol absorption [55].

Dairy products contain antioxidant compounds such as sulfur amino acids, whey proteins such as β lactoglobulin, and essential vitamins such as A, E, and C. These antioxidants, along with microelements such as Sn, Zn, Fe, and Mn, found in milk and dairy products, help to improve the antioxidant capacity to combat stress. Proteins play a role in enhancing the antioxidant capacity of these products (Table 2). Additionally, bioactive peptides [8,68,74,75] released during fermentation or cheese aging contribute to the antioxidant activity of dairy products. The inhibition of dipeptidyl IV (DPP-IV) and the human insulin receptor (hIR) were observed in vitro as the way of action of whey proteins and hydrolysates of milk [69]. To boost the content of metabolites in milk and dairy derivatives, animal nutrition practices and natural additives, such as mixtures, seeds (*Acacia*), fruits (mulberry), and byproducts from the fruit and vegetable industry (black rice, purple corn residue, grape residues, essential oil from orange, palm seeds), can be utilized. Practices, such as grazing, have been proven to increase antioxidants in milk. Moreover, incorporating strains such as *Lactobacillus acidophilus* is a recognized method to elevate antioxidant levels. Thus, by adding ingredients to animal feed or during the production of milk, we can enhance the properties of these products. Consuming dairy products with antioxidants not only reduces the chances of lifestyle chronic-related illnesses but also the course of aging [2,12,76].

Carotenoids are a chemical group of metabolites found in plants and other living organisms such as bacteria, fungi, and algae. These natural components are pigments (red, yellow, and orange colors), which can be divided into: carotenes, which contain solely carbon and hydrogen in their structures; and xanthophylls that, additionally to carbon and hydrogen, include oxygen. Carotenoids are lipophilic compounds and are therefore mainly stored within the fatty share of the non-liquid proportion of milk from ruminants of all species. Though there is a wide range of carotenoids, only a few of them are normally identified in milk with β-carotene (up to 75–90% of the total content in milk) and lutein, being the two most abundant compounds of this chemical group. Some other carotenoids may be present (e.g., zeaxanthin), but its occurrence varies largely across research studies. It has been indicated that the levels of β-carotene and lutein can differ depending on the type of forage consumed by animals. For instance, a mixture of temperate forages containing clover showed significantly higher concentrations of β-carotene and lutein (235 and 655 mg/kg dry matter, respectively) compared to a forage mixture containing predominantly lucerne (148 and 433 mg/kg dry matter, respectively). In the same line, preserved forages typically have lower carotenoid levels compared to fresh grass-based diets. The phenological stage of the forages may also affect its carotenoid content. For example, during the growth phase of mountain forages, there was a decline in carotenoid concentration (from 5.4 to 3.9 µg/g of fat), followed by an increase during regrowth (reaching 4.9 µg/g). On the other hand, animal metabolism may also affect the carotenoid content of milk. In a trial performed on sheep, a high initial concentration of carotenoids was observed in the liquid of rumen, with a subsequent increase of lutein, 13-cis-β-carotene, and trans-β-carotene (174, 17 and 21 mg/day) levels after post-ruminal digestion. Further, an increment of these compounds was observed in the duodenum (204, 54, and 64 mg/day, respectively). This is possible because of the novo synthesis of carotenoids in the rumen. However, if plasma carotenoid concentrations in the ruminants reach close to 5 µg/mL, a metabolic saturation of β-carotene occurs, and further intake of carotenoids does not translate into a significant raise in the concentration of such metabolites in milk [77].

Ali and colleagues [78] conducted a review exploring the health benefits and functional characteristics of peptides from milk proteins. These peptides are highlighted as components that do not only boost the nutritional value of dairy products but also contribute to their health-promoting qualities. Some of the peptides found in milk include casomorphins, α lactorphins, β lactorphins, lactoferroxins, casoplatelins, casoxins, casokinins, and lactoferricin. These peptides prove bioactivities such as opioid agonists, inhibition effects of the angiotensin-converting enzyme, immunomodulatory properties, and antimicrobial activities. The implications of these findings suggest that incorporating milk-derived peptides into food products can provide potential health benefits to consumers. To enhance the health potential of dairy byproducts, the authors recommended utilizing natural resource extracts, e.g., seaweed, stevia, moringa, strawberry, rice, green tea, among others. Their bioactive compounds can contribute to overall health advantages [75,79]. Therefore, integrating milk-derived peptides offers an opportunity to amplify the nutritional value and health-enhancing characteristics of dairy products.

Grazing animals on pastures not only provides nutritional advantages but also contributes to preserving plant diversity by preventing the disappearance of certain plant species and improving their abundance of others [80]. Moreover, harmful levels of certain metabolites are kept below critical levels of ingestion [7]. For example, leguminous plants contain condensed tannins that can boost milk and wool output, enhance lambing rates, and lower the chances of bloating and intestinal parasites [3]. Furthermore, legumes rich in tannins can reduce methane production in the rumen, thereby cutting down on greenhouse gas emissions and energy loss during digestion [3,20,81]. Despite some uncertainties surrounding productivity, diverse grasslands offer a range of benefits such as improved product quality, animal wellbeing, nutrient preservation, and stable production [82], all in the face of changing climate conditions.

Ruminants grazing on diverse pastures, compared to indoor feeding with concentrate or install diets, markedly alters the fatty acid composition in animal products [72,80]. Specifically, grazing increases the levels of beneficial unsaturated fatty acids, such as linoleic and linolenic acids, which not only improve the sensory qualities and shelf-life of meat but also contribute to a darker coloration [83]. This has been observed on different animal species, seasons, and locations [37]. Research indicates that dairy products from grazing ruminants contain higher concentrations of these unsaturated fatty acids, thereby enhancing their health benefits, compared to products from animals largely fed on concentrates. Grazing and browsing practices have proven to be effective in enhancing the bioactivity of milk and cheese, providing a sustainable production method that aligns with natural resource availability and animal needs [73]. Both aspects must be considered to assure a successful intervention on animal farming aims. This approach not only supports food security but also increases the income of smallholders globally. Most diverse pastures are rich in plant bioactive compounds, which are superior to those from monocultures or low-species grasslands [84]. For example, plantain can improve the fatty acid profile of cow milk, with a higher content of polyunsaturated fatty acids (+92%), omega-3 (+101%), and 6 (+113%), 4-methylcatechol sulfate, and p-cresol glucuronide in relation to milk coming from cows fed with ryegrass [75].

In regions where goats graze on biodiverse pastures, despite facing challenges such as low quality forage and limited water supply, goat milk and cheese are known for their health benefits [72,73,83]. Goat farming systems in developing countries encounter obstacles such as forage and water scarcity; yet, they have the potential to produce dairy products with health-promoting properties [85]. This is possible because the bioactive substances present in the animal diets and subsequently in these dairy products, including polyunsaturated fatty acids, catechins, and phenolic acids, are associated with antioxidant activity and the modulation of chronic diseases in mice [37]. The influence of animal nutrition on milk and cheese impact has been studied, showing significant advantages from diets containing abundant bioactive compounds. These plant-based nutrients possess inflammatory, antioxidant, anticancer, and heart-protective attributes that contribute to the health advantages of milk and its derivatives [86,87].

A detailed analysis of the botanical components consumed by grazing animals and the metabolic transformations is essential to understanding the complete process of plant bioactive compounds [87]. Bridging these knowledge gaps will help us better understand how to enhance the occurrence and concentration of beneficial compounds in animal-derived foods. Also, incorporating diverse plant species into grazing systems supports sustainable animal production and enriches the nutritional and bioactive profile of animal products. This approach has significant implications for food security, the economic stability of smallholders, and public health, making it a vital area of ongoing research and practical application. The integration of bioactive compounds from plant-based diets through grazing and browsing not only improves the nutritional quality of animal products but also contributes to sustainable livestock farming, promising better health outcomes for both animals and humans.

**Table 2:** Metabolites and health benefits and implications of milk and dairy by-products.

Metabolite Category	Metabolite	Feedstuffs or Dairy Product	Dose, Concentration, or Treatment	Biological Function of Metabolites, Biochemistry, and Biotransformation	Reference
Phenols	Tannin	Blood plasma and cheese	Control group and Tannin group supplemented with 150 g/head per day of tannins extract, chestnut and quebracho (60:40).	Serum: tannin supplementation lowered oxidative stress both in spring and in summer. Lowered oxidative stress (IL-1β and higher IL-10). Cheese: improvement of the antioxidant properties.	Santillo et al., 2022 [10]
Bioactive peptides	Bioactive peptides	Hydrolysates of camel milk	Hydrolysates at 500 mg/kg of BW	Antidiabetic properties. Hypoglycemic activity and improvement in activity of superoxide dismutase and catalase. Reduced glutathione levels and the attenuation of malondialdehyde. Lower levels of liver function enzymes (aspartate aminotransferase and alanine aminotransferase). Histology of liver and pancreatic tissue displayed absence of lipid accumulation in hepatocytes and preservation of β-cells.	Kilari et al., 2021 [88]
Camel whey proteins	Camel whey proteins	Camel milk	Hydrolysates at 500 mg/kg of BW	Antidiabetic properties. Inhibition of DPP-IV (dipeptidyl peptidase IV) and their positive action on hIR (the human insulin receptor) activation and glucose uptake.	Ashraf et al., 2021 [89]
Phenolic acids	Anthocyanin	Black rice and purple corn extracted residue	0, 2, 4, and 6% black rice and purple corn extracted residue	Increase of antioxidant activity and reduction of oxidative stress in plasma. Malondialdehyde (MDA) concentrations in the plasma decreased.	Prommachart, et al., 2021 [90]
Phenolic acids	Grape seed and grape marc meal extract	Grape seed and grape marc meal extract	0, 1% of grape seed and grape marc meal extract or the same total mixed ration supplemented with	Cows fed grape seed and grape marc meal extract had an increased milk and protein yield. Reduced mRNA presence of fibroblast growth factor (FGF) 21.	He et al., 2019 [91]
Phenolic acids	Grape seed and grape marc meal extract	Grape seed and grape marc meal extract	1% grape seed and grape marc meal extract	Cows supplemented with grape seed and grape marc meal extract had a significantly reduced mRNA abundance of fibroblast growth factor (FGF) 21.	Gessner et al., 2015 [92]
Phenolic acids		Grape residue silage in the diet	Grape residue silage (0, 50, 75, 100 g/kg DM)	Antioxidant activity in milk was higher with increased dietary levels of grape residue silage.	Santos et al., 2014 [93]
Phenolic acids	Total phenols, total tannins, condensed tannins	Weed species as additives	Tithonia tubiformis (5% of inclusion in the diet of sheep)	Inclusion of *T. lucida* in the sheep diet resulted in an increase in total phenol content (18%) and an increase in antioxidant activity (30%)	Diaz-Medina et al., 2021 [94]
Phenolic acids	Total phenols, total tannins, condensed tannins	Dried by-products	100 g/day per head of tomato pomace 100 g/day per head of grape marc 75 g/day per head of exhausted myrtle berries	Dried by-products increased antioxidant activity in milk and blood plasma of dairy ewes. Grape marc elevated C18:2n-6.	Buffa et al., 2020 [95]
Phenolic acids	Hydrocinnamic acids, flavonoids	Goat milk cheeses	Grazing versus indoor feeding	Grazing feeding increases the quantitative and qualitative antioxidant activity of goats’ milk cheese. Also, the content of some metabolites, such as hydrocinnamic acid, were increased.	Cuchillo et al., 2010 [72]
Fatty acids	Fatty acids	Goat milk cheeses	Grazing versus indoor feeding	Major polyunsaturated fatty acids in milk and cheese from goats were increased by grazing compared to indoor systems.	Cuchillo et al., 2010 [73]
Phenolic acids	Gallic, caffeic, chlorogenic, and ferulic acids, catechin, epicatechin, and quercetin	Goat milk	Inclusion of *Acacia* *farnesiana* pods meal in goat diets	*A. farnesiana* increased the presence of bioactive compounds and the antioxidant activity of goats’ milk, while cholesterol content was reduced.	Delgadillo-Puga et al., 2019 [83]
Bioactive compounds	Fatty acids, monoterpene and sesquiterpene, tocopherol, linoleic acid	Cow and goat milk cheeses	Grazing versus indoor feeding	Grazing feeding increases terpenes, tocopherol, and antioxidant activity of cow and goat milk cheeses. Fat and cholesterol contents were diminished.	Galina et al., 2007 [85]
Bioactive compounds	Polyphenol, hydroxycinnamic acids, flavonoids, fatty acids.	Goat milk and cheeses	Grazing versus indoor feeding	Grazing/browsing promote the transference of bioactive compounds from vegetation to animal products. Supplementation with rich-bioactive compound forages increased the bioactive compounds in milk and cheese. The consumption of goat milk prevents obesity, insulin resistance, inflammation, and hepatic steatosis in mice.	Delgadillo-Puga and Cuchillo-Hilario, 2021 [37]
Bioactive compounds	Polyphenol, hydroxycinnamic acids, flavonoids, and fatty acids.	Goat milk and cheeses	Grazing versus indoor feeding	Goat milk intake prevents obesity, reduces fat mass, and increases lean mass in the mice fed with high fat diets. Also, there was a reduction in inflammatory markers, an increase in energy expenditure, and a higher presence of mitochondrial content in the skeletal muscle of mice.	Delgadillo-Puga et al., 2020 [86]
Terpenes	d-limonene (95.17 g/100 g orange peel essential oil)	Blood plasma and milk antioxidant activity.	Dietary orange peel essential oil inclusion in lactating dairy ewes’	Inclusion of 300 mg of orange peel essential oil/kg to ewes increased milk saturated fatty acids. Addition of 450 mg of orange peel essential oil/kg to ewes concentrate improved blood plasma and milk antioxidant activity.	Kotsampasi et al., 2018 [96]
Phenolic acids	Anthocyanins	Blood plasma and milk of goats	Anthocyanin-rich purple corn stover silage on goats feeding	Lactating goats fed with anthocyanin-rich purple corn stover silage resulted in higher levels of peonidin and malvidin-3-O-glucoside and a higher level of superoxide dismutase (SOD) in plasma and milk relative to the control diet.	Tian et al., 2019 [97]
Phenolic acids	Not determined	Blood plasma and milk of goats	0% (control), 6%, 12%, and 18% of date palm (*Phoenix dactylifera* L.) seed	Date palm increased antioxidant capacity in milk and blood of dairy goats. Conjugated linoleic acid (CLA) in milk was also increased.	Sharif et al., 2017 [98]
Essential oils	Carvacrol, p-Cymene, Borneol, Β-Caryophyllene	Blood plasma and milk of goats	Thirty g equivalent to a daily dosage of 1 mL of essential oil of *Origanum vulgare* ssp. Hirtum. per animal.	*Origanum vulgare* increases the glutathione peroxidase and glutathione reductase both in blood and milk.	Paraskevakis et al., 2015 [99]

### 4.2. Meat and Meat By-Products

Meat as the part of an animal includes muscle tissue, viscera, and blood. Marine mammals are also considered meat in terms of cultural diet [100]. Meat products can be categorized as fresh or processed meat. Muscle requires a maturation process to obtain meat. Chemical composition can vary depending on species, breed, age, feeding management, and retail cuts. Sixty percent of the raw weight of the muscle is water, while carbohydrates such as glycogen are in reduced amounts (except in the liver). Protein ranges from 17% to 22%. Meat proteins are valuable because of their high digestibility and their essential amino acid content [101]. Fat content varies among animal species and is influenced by specific anatomical locations. Lean or low-fat meat content is 10% of fat (USDA, 2020). Red beef meat is an important source of B vitamins, zinc, and iron minerals. Meat and meat by-products are rich in saturated fatty acids (SFA). The increase in the consumption of SFA is correlated with the development of cancer and cardiovascular disease (CVD) risks. A decline of nearly 14% in beef red meat consumption has been reported by the European Environment Agency (2017) from 2000 to 2023. Also, the Dietary Guidelines for Americans (2020–2025) suggest the consumption of monounsaturated fatty acids and polyunsaturated fatty acids in substitution of SFA [102]. Another strategy to improve the bioactive compounds in beef meat is the influence of pasture feeding; recently, Stanton et al. [103] reviewed the influence of pasture feeding on meat products in terms of human health and product quality, highlighting the clinical trials of Gilmore et al. [104] and Adams et al. [105], who evaluated beef meat as a result of animal feeding system on cholesterol; Gilmore et al. [104] incorporate 114 g ground beef patties/weeks for 5 weeks derived from pasture-fed cattle (low MUFA) or grain-fed cattle (high MUFA). The intervention derived from grain-fed cattle significantly increased HDL cholesterol from the baseline. A clinical trial was developed with 10 men who consumed hamburger patties derived from pasture-fed cattle (MUFA: SFA = 0.95; high SFA) during 5 weeks, after a 3 weeks washout period, they consumed hamburger patties from grain-fed cattle (MUFA: SFA = 1.31; high MUFA) for another 5 weeks. Following consumption of the high SFA hamburger; the values of plasma triacylglycerols and LDL:HDL ratio were significantly higher than after the high MUFA hamburger phase. Conversely, HDL cholesterol was greater after the high MUFA hamburger phase than after the high SFA hamburger phase. These studies used meat of the feedlot system at Adams et al. [105]. Provenza et al. [106] questioned whether grass-fed meat is better for human health and the environment. They mentioned that health is enhanced when livestock forage comes from grasslands with high plant biodiversity since they are commonly rich in phytochemicals. Conversely, when livestock forage comes from a simple mixture of forages or from monoculture pastures or cattle consuming high-grain rations in feedlots, animals’ and humans’ health is rarely improved. Grass-based diets can either reduce the ecological impact of livestock farming or provide forages rich in phytochemicals to the cattle, such as polyphenols, and transfer them as their secondary compounds to animal products, causing a positive effect on consumers’ health [107].

Meat products are derived from processing muscle and fat as the primary ingredients. In addition to these main components, a diverse array of non-meat substances is utilized in producing processed meat products. Essential substances such as salt and seasonings are employed alongside others, specific to individual products. Technological treatments such as grinding, drying, heating, curing, or fermentation produce semi-processed or processed meat products [100]. Enhancing these with functional ingredients from a technological standpoint to elevate both technological and sensory characteristics has been a constant in the meat industry. The use of functional ingredients during the processing of meat by-products includes vegetal and animal protein. To elaborate meatballs, burgers, sausages with pork, beef, and lamb meat are combined with added soy flour, chickpea and green lentil flours, wheat gluten, soy protein isolate, sodium caseinate, or whey protein isolate, rye bran, egg yolk, egg white, oat bran, inulin, pectin, tomato fiber pectin, barley fiber, and other ingredients. These all have the main goal of improving firmness, consistency, sensory and texture properties, as well as softer texture, and reducing cooking and frying loss [108,109,110,111,112,113,114].

Health-promoting compounds such as spices, essential oils, vegetables, fruits, waste, and grass-fed meat and their meat by-products have been developed by food technology. In this way, carotenoids, fatty acids, fiber, essential oils, phenolics, and polyphenols (such as flavonoids) have potential health benefits like anti-inflammatory, anti-carcinogenic, and antioxidant effects. Other beneficial substances in meat and meat by-products such as dried fermented sausage, salami, chorizo, pepperoni, and others are probiotics, bioactive peptides, and antioxidants. More research is needed to fully understand how livestock production practices impact the health benefits of animal foods for humans (Table 3).

Fruits, herbs, spices, and vegetable extracts are sources of natural antioxidants and are alternatives to traditional synthetic antioxidants. Additionally, several studies show the effectiveness of natural antioxidant supressing lipid oxidation in meat and meat by-products without affecting their taste and smell. In this way, Galli et al. [115] added curcumin microencapsulated as a supplement to red meat. The presence of curcumin, carvacrol, thymol, and cinnamaldehyde improves meat quality, increases antioxidant levels and reduces lipid peroxidation. Hassan et al. [116] added grape seed extract (GSE) as a non-conventional resource that was added to rabbit feed. Grape seed extract metabolites contain phenolic acid, anthocyanins, flavonoids, and monomeric phenolic compounds, such as (+)-catechins, (−)-epicatechin, and (−)-epicatechin-3-O-flattened dimeric, trimeric, and turmeric procyanidins, and condensed tannins. These were biotransformed to improve the activity of antioxidants in rabbit blood; antioxidant enzymes (superoxide dismutase, catalase, glutathione peroxidase, and glutathione transferase) activity and total antioxidant capacity in blood were increased (*p* ≤ 0.05) by adding dietary GSE.

Tannin sources were added to rabbit feed (0.5% and 1%) using chestnut wood extract (CWE) and *Castanea sativa* without any adverse effects on the carcass and meat traits of rabbits. Concerning the antioxidant effect, CWE showed effects at the inclusion level of 0.5% but showed a pro-oxidant outcome at 1% inclusion. CWE had no practical effect on the fatty acid profile of rabbit meat [117]. Essential oils of spices such as oregano (*Origanum vulgare* L.) and sweet chestnut vegetable extract are good sources of bioactive compounds [118]. Ranucci et al. [119] and Fasseas et al. [120] reported the presence of carvacrol 5.8%, thymol 60.9%, p-cymene 10.5%, linalool, phenolic acids, flavonoids, and triterpenoids (γ-terpinene 7.6%). These authors reported that these essential oils improved the oxidative stability of sheep, pork, and beef meat produced; which may positively impact consumer health. As well, Fasseas et al. [120] reported essential oils: eucalyptol 49.4%, camphor 8.5%, and α-pinene 5.4% in *Salvia officinalis,* and other essential oils were added to beef and pork meat to reduce oxidation.

The hydroxybenzoic acids, hydroxycinnamic acids, and flavonoids present in the oregano extract were reported by Fernandes et al. [121], who recommended 24 mL of oregano extract/kg as a natural antioxidant in lamb burgers. Additionally, they also evaluated the oregano extract and found it had antioxidant potential equivalent to sodium erythorbate at intermediate (6630.98 mg/kg) and high (8038.20 mg/kg) levels, calculated by DPPH∙ and FRAP methods, allowing synthetic antioxidants to be replaced while maintaining the nutritional and sensory quality of cooked sheep sausages.

Wine-making by-product meal (WBM), other non-conventional polyphenols, and antioxidant sources were analyzed by de Alencar et al. [122]. They evaluated WBM extract by HPLC and found 25 phenolic compounds, one of which was below the limit of quantification (procyanidin A2), totaling 9.51 mg phenolic compounds per gram of extract. The flavonol group was the major group (36.4%), followed by anthocyanins and tannins with 24.07% and 13.5%, respectively. WBM is a natural antioxidant that replaces butylhydroxytoluene (BHT) in beef burgers stored at −20 °C for up to 120 days. WBM can be used as a natural antioxidant to replace BHT in preparing beef burgers stored at freezing temperatures, at a maximum content of 1 g/100 g of product. Higher WBM levels increased lipid oxidation and decreased product sensory quality. Cooking losses were also higher when WBM was used at higher levels (1.5 and 2 g/100 g). At all levels of WBM inclusion, beef burgers had a crude fiber content of >3 g/100 g of product, which is enough for them to be labeled as sources of dietary fiber.

On the other hand, Ahmad et al. [123] employed plum puree, prunes (dried plum), and plum extracts as polyphenols; 3% plum extract treatment had a reduced (*p* < 0.05) TBARS value of 0.84 mg MDA/kg meat after 7 days of storage at 4 °C. Additionally, Núñez de González et al. [124] added 2.5% or 5% of a fresh plum juice concentrate to raw and precooked pork sausages, showing a reduction of TBARS values, inhibiting lipid oxidation, and having minimal effects on tenderness, sensory characteristics, color, and appearance.

As previously mentioned, fiber is an excellent health option for consumers. Acceptance of healthy foods and food neophobia (defined as “fear of trying new foods”) showed that there is potential for consuming new formulations in cooked sausages with functional ingredients, such as dietary fiber (cactus pear fiber or pineapple fiber). In this way, Díaz-Vela et al. [125] evaluated how the addition of 2% of cactus pear (*Opuntia ficus indica*) or pineapple (*Ananas comosus*) impacts the attributes such as color, sweet, astringent, and bitter flavors, pork meat smell, and firm and plastic texture. Chaparro et al. [126] also added orange peel flour or maguey leaf fiber (3%) to sausages. One hundred and fifty participants tasted sausages with orange peel flour and maguey describing significantly higher levels of bitter, astringent, and spicy notes than the control.

Pomegranate peel flour is a functional ingredient that can be employed to replace part of the fat in a raw meat product such as chorizo, since the polyphenols and dietary fiber content improve texture and enhance coloration, helping in product shelf life (decreasing water activity and promoting lower pH). Maillard-Berdeja et al. [127] added 2% or 4% pomegranate peel flour to chorizo, resulting in a more rigid texture, a crumbly consistency, and a less intense color. The search for new alternatives does not always result in products pleasing to the palate of all consumers. Challenging new components is a task of continuous research and innovation.

As part of the ongoing efforts to enhance the accessibility of bioactive compounds such as probiotics to the general consumer, various propositions have been introduced, such as global fermented foods, including incorporating *Lactobacillus (LAB): Lb. plantarum, Lb. paraplantarum, Lb. brevis, Lb. rhamnosus, Lb. sakei, Lb. zeae, Lb. paracasei, Lb. sake, Lb. curvatus, Lb. plantarum, Ent. faecalis, Ent. faecium, Leuconostoc mesenteroides, Pediococcu pentosaceus, Ped. acidilactici, W. cibaria,* and *W. viridescens* in dried fermented sausage (salami, salsiccia, soppressata, alheiras, botillo, chorizo, salchicón, pepperoni) [120,121]. Hernández-Alcántara et al. [128] evaluated *Enterococcus faecium* (UAM1 strain) and five *Pediococcus pentosaceus* (UAM2-UAM6) strains. They analyzed the auto-aggregation ability of *Lactobacillus*, where six thermotolerant lactic acid bacteria were isolated from cooked meat products such as Vienna sausages. Additionally, they evaluated the adherence of *E. faecium* UAM1 to the human epithelial cell line Caco-2 (around 20%), and it was significantly higher than that obtained with the *P. pentosaceus* strains (2–5%) and *Lactobacillus acidophilus* LA-5 (6%). The overall results indicate that *E. faecium* UAM1 has activity as a probiotic.

Another essential aspect of the meat industry is the creation of functional components during processing; bioactive peptides are organic substances formed by amino acids joined by covalent bonds known as amide or peptide bonds [129]. The bioactive peptides can be produced from precursor proteins in three ways: (a) enzymatic hydrolysis by enzymes extracted from microorganisms or plants; (b) enzymatic hydrolysis by digestive enzymes; (c) fermentation by proteolytic starter cultures, in most studies, a combination of (a) and (b) or (a) and (c) has been effective in the production of short-chain peptides. The composition and sequence of the amino acids determine their different functions, including relaxing effects, solute binding properties, strengthening of the immune system, antioxidant, anti-microbial, anti-inflammatory, cholesterol-lowering, and anti-hypertensive effects [130,131]. In this way, meat and meat by-products are important sources of bioactive peptides. López-Pedrouso et al. [132] employed the porcine liver as pigments and bioactive peptides and evaluated two isoforms of aldehyde dehydrogenase (I3LRS5, ALDH1A1) and four peptides from fructose bisphosphate aldolase (A0A4X1VHB8, ALDOB) as correlated with antioxidant and antihypertensive elements. Mora et al. [133] reported that Iberian dry-cured ham is a source of different bioactive peptides due to its high protein content and intense hydrolysis during its processing; the bioactive peptides in Spanish dry-cured ham are AEEEYPDL and LGVGG, which have been reported as α-glucosidase inhibitory activity. This enzyme is frequently used as a target for the treatment of type 2 diabetes mellitus (T2DM). A few years later, Gallego et al. [134] evaluated the dipeptides and peptides from Spanish dry-cured ham extracts. Peptides KA and AAATP showed DPP-IV inhibitory activity, with IC50 values of 6.27 mM and 6.47 mM, respectively. The authors suggest this ham is as a natural potential precursor of DPP-IV inhibitory peptides which could be used against type 2 diabetes. Martini et al. [135] also reported the ability of pork meat on dipeptidyl peptidase IV (DPPV-IV) activity inhibition and their regulation of T2DM. These bioactive peptides affect the activity of various enzymes involved in carbohydrate metabolism, insulin secretion, and the release of incretin hormones such as glucose-dependent insulinotropic peptide (GIP) and glucagon-such as peptide-1 (GLP-1) that affect postprandial blood glucose levels. Kęska and Stadnik [136] also reported the dipeptidyl Peptidase IV inhibitory peptides generated in dry-cured pork loin during aging; the sequences APPPPAEV, APPPPAEVH, KLPPLPL, RLPLLP, VATPPPPPPK, VPIPVPLPM, and VPLPVPVPI show promise as natural food compounds helpful in maintaining good health. Antibiotical properties of the bioactive peptides of blood as a meat by-product were documented by Abou-Diab et al. [137]; a MIC value of 0.31 mg/mL inhibited *S. aureus*.

Chicken slaughterhouse by-products can create protein hydrolysates with ACE-inhibitory activity, lowering blood pressure and improving endothelial dysfunction [138]. A recent study by Ibarz-Blanch et al. [139] mentioned that these hydrolysates maintain important biological activities as antioxidant, anti-inflammatory, anti-coagulant, anti-anaemic, cardioprotective, hepatoprotective, and neuroprotective agent. Further clinical studies are needed to confirm their properties and contribute to the circular economy model in slaughterhouses.

Natural bioactive compounds of meat, such as carnosine, have been characterized as antioxidants; carnosine can neutralize and reduce oxidative reactivity. This compound has been described as an antioxidant in chicken, red meat, and processed meat. Carnosine is a dipeptide composed of Beta-alanine and L-histidine, which is found mainly in muscles and brain. This suggests that radical scavenging ability can mainly be attributed to the N-terminus on the L-histidine residue (the imidazole ring); non-histidine-containing amino acids have limited interaction with the aldehydes [140,141]. Carnosine obtained from chicken, red meat, and processed meat has also been evaluated as potentially beneficial in healthy and diseased myocardial models [142].

The oxidation of long-fatty acids into the mitochondria requires the binding of carnitine to form acylcarnitines, which play a vital role in fatty acid metabolism. Studies suggest that acetylcarnitines can have a significant impact on mental health and brain function [143,144]. Fish and red meat are other important bioactive sources of acylcarnitines:acetyl-carnitine, propionyl carnitine, and 2-2-dimethyl butyryl carnitine. McCann et al. [145], showed that L-carnitine and acylcarnitines are targets of mitochondrial biomarkers for precision medicine.

Increased investment, comprehensive research, and innovative strategies are essential for developing new products tailored to meet consumer needs, to ensure acceptability, and to positively impact health. This approach aims to shift the prevailing negative perception of meat products.

**Table 3:** Metabolites with health benefits in meat and meat by-products.

Category	Metabolite	Meat Product or By-Product Added to Feedstuff	Dose, Concentration, or Treatment	Biological Function of Metabolites, Biochemistry, and Biotransformation	Reference
Acyl-coenzyme A (CoAs)	Acylcarnitines: acetylcarnitine, propionylcarnitine, and 2-methylbutyrylcarnitine	Meat and fish meat	NR	The transportation of long-chain fatty acids into mitochondria requires carnitine to form acylcarnitines, which is also suggested to impact mental health and brain function.	Cheung et al., 2017; Li et al., 2019 [143,144]
Amino acids (AA) biomarkers	Urinary carnosine, 1-methylhistidine and 3-methylhistidine.	Meat	1.5 g/kg/d	In the meat protein-based diet, the main protein sources were pork, beef, and chicken. Urinary and plasma AA may be potentially useful biomarkers for meat protein intake.	Altorf-van der Kuil et al., 2013 [146]
Antioxidants	Phenolic acids, anthocyanins, flavonoids, monomeric phenolic compounds such as (+)-catechins and (−)-epicatechin, as well as dimeric, trimeric, and turmeric procyanidins and condensed tannins. ⤉	Grape Seed Extract (GSE)	100 and 300 mg GSE/kg	Antioxidant enzymes of rabbits (superoxide dismutase, catalase, glutathione peroxidase, glutathione transferase) and total antioxidant capacity in blood were increased (*p* ≤ 0.05) by adding dietary GSE.	Hassan et al., 2016 [116]
Antioxidants	Carnosine	Chicken, red meat, processed meat	NR	Carnosine, a dipeptide of Beta-alanine and L-histidine found in muscles and the brain, exhibits radical scavenging ability primarily due to the imidazole ring on the L-histidine residue.	Boldyrev 1993; Zhou et al., 1999 [140,141]
Antioxidants	Gallic acid; catechin and epicatechin. ⤉	Wine making by-product meal (WBM)	0.5, 1 and 2%	WBM can replace butylhydroxytoluene (BHT) as a natural antioxidant in beef burgers stored at −20 °C for up to 120 days, at a maximum of 1 g/100 g. Higher WBM levels increase lipid oxidation and decrease sensory quality, while all levels provide enough crude fiber (>3 g/100 g) for dietary fiber labeling.	de Alencar et al., 2022 [122]
Antioxidants	Phenolic acids: gallic and protocatechuic acids; Flavanols: catechin, epicatechin, and proanthocyanidin B1 Flavonols: quercetin 3-O-rutinoside, quercetin 3-O-glucoside, and kaempferol 3-O-glucoside. ⤉	Wine making by-product meal (WBM)	Gallic acid 16.66 mg/100 g 29, 33, and 40 mg/100 g of catechin, epicatechin and proanthocyanidin B1, respectively. Quercetin 3-O-glucoside (48.8 mg/100 g)	Winemaking by-products represent a source of phenolic compounds with antioxidant and anti-cholinesterase activities.	Jara-Palacios et al., 2020 [147]
Antioxidants	NR	Fresh plum juice concentrate (FP)	2.5 and 5%	All plum ingredients reduced TBARS values, inhibited lipid oxidation, and minimally affected tenderness, sensory characteristics, color, and appearance in raw and precooked pork sausage.	Nuñez de Gonzalez et al., 2008 [124]
Antioxidants	Hydroxybenzoic acids, hydroxycinnamic acids, flavonoids. ⤉	*Origanum vulgare* extract	13.3, 17.8, and 24.0 mL/kg	24 mL/kg of oregano extract could be recommended as a natural antioxidant.	Fernandes et al., 2017 [121]
Antioxidants	Carvacrol, thymol, p-cymene, rosmarinic acid, α-thujene, α, β-pinene, p-coumaric acid, and γ-terpinene. ⤉	*Origanum vulgare* extract		Protein oxidation is initiated by myoglobin, metallic catalysts, or oxidizing lipids reacting with amino acid side chains, leading to carbonyl derivatives and protein carbonylation, or radicals.	Ranucci et al., 2015 [119]
Antioxidants	NR	*Origanum vulgare* extract ⤉	4964, 6630, and 8038 mg/kg	The extract showed antioxidant potential similar to sodium erythorbate at intermediate and high levels, measured by DPPH and FRAP methods.	Fernandes et al., 2017 [121]
Bioactive peptides	Neokyotorphyn (alpha 137–141) TSKYR.	Meat by-products: Blood	0.31 mg/mL	Antibiotical properties with an MIC value of 0.31 mg/mL for *S. aureus*.	Abou-Diab et al., 2020 [137]
Bioactive peptides	I3LRS5, ALDH1A1, A0A4X1VHB8, and ALDOB	Porcine liver protein fraction at pH 4.8 showed antioxidant capacity but antihypertensive inhibition	NR	Two isoforms of aldehyde dehydrogenase (I3LRS5, ALDH1A1) and four peptides from fructose bisphosphate aldolase (A0A4X1VHB8, ALDOB) were correlated with antioxidant and antihypertensive activities.	López-Pedrouso et al., 2023 [132]
Bioactive peptides	AEEEYPDL and LGVGG	Iberian dry-cured ham, proteins that are hydrolyzed during processing, is a source of bioactive peptides.	NR	Bioactive peptides in Spanish dry-cured ham have AEEEYPDL and LGVGG α-glucosidase inhibitory activity.	Mora et al., 2020 [133]
Bioactive peptides	KA and AAATP	Spanish dry-cured ham extract (SD-CHE)	IC50 6.27 mM and 6.47 mM	Peptides KA and AAATP from SD-CHE exhibit strong DPP-IV inhibitory activity (IC50 values: 6.27 mM and 6.47 mM), indicating their potential for functional products targeting type 2 diabetes.	Gallego et al., 2014 [134]
Dipeptide	Carnosine is a pleiotropic histidine-containing dipeptide synthesized from β-alanine and L-histidine	Meat chicken, red meat, processed meat	8.0 ± 4.3 μg/g	Carnosine in myocardial tissue is promising, potentially beneficial in both healthy and diseased myocardial models.	Creighton [142] et al., 2022 [142]
Essential Oils	Hymol 60.9%, p-cymene 10.5%, γ-terpinene 7.6%, and carvacrol 5.8%. ⤉	Oregano essential oil of *Origanum vulgare* L.	1 mL/kg	Improved the oxidative stability of the sheep meat produced.	Simitzis et al., 2008 [118]
Essential Oils	Carvacrol, thymol, p-cymene, linalool, phenolic acids, flavonoids, and triterpenoids. ⤉	Oregano essential oil and sweet chestnut wood extract.	0.2%	Improved the oxidative stability of the pork meat produced.	Ranucci et al., 2015 [119]
Essential Oils	Oregano essential oils (OEO) extract. ⤉	Oregano essential oil	130 and 230 mg/d OEO	Dietary with OEO increases antioxidant capacity and enzyme activities and reduces pH, cooking loss, and malondialdehyde content. It also enhanced polyunsaturated fatty acids, conjugated linoleic acid, and essential amino acids in the Longissimus thoracis muscle.	Jara-Palacios [147] et al., 2020 [147]
Essential Oils	Oregano essential oil: thymol 60.9%, p-cymene 10.5%, γ-terpinene 7.6%, and carvacrol 5.8%. Sage essential oil: eucalyptol 49.4%, camphor 8.5% and α-pinene 5.4%. ⤉	*Origanum majorana*, *Origanum vulgare*, and *Salvia officilalis*	3%	Meat beef and pork essential oil treatments significantly reduced the oxidation.	Fasseas et al., 2008 [120]
Fatty acids	Stearic acid and oleic acid ⤉	Rambouillet lambs	50 and 100 g	Including chia seeds in lambs’ diets increased the bodyweight of neither the meat carcasses nor the non-meat components. It tended to increase the oleic acid and decrease the stearic acid in the meat.	Uribe-Martínez et al., 2023 [148]
Fiber	NR	Goat meat	25 and 55%	Consumers preferred meat from kids fed a diet with 55% forage cactus, which resulted in lower lipid content and higher levels of monounsaturated fatty acids in goat meat.	Pinheiro et al., 2023 [149]
Fiber	Linoleic, conjugated linoleic, eicosapentaenoic, docosahexaenoic ⤉	Lamb meat	≤28 g/kg DM	Microalgae Spirullina platensis, Schizochytrium sp.MIA supplementation increased (*p* < 0.05) the content of linoleic, conjugated linoleic, eicosapentaenoic, docosahexaenoic, and total ω-3 FAs in meat.	Orzuna-Orzuna et al., 2023 [150]
Fiber	NR	Lamb meat	2 and 4 g/kg DM	Ddetected better animal performance and physicochemical characteristics in meat from lambs supplemented with microalgae	Alghonaim et al., 2022 [151]
Fiber	NR	Sausages with orange peel flour or maguey leaf (SWOPFML)	3%	150 people were assessed to taste SWOPFML, which had significantly higher levels of bitter, astringent, and spicy notes than the control.	Chaparro et al., 2013 [126]
Fiber	NR	Cooked sausage	2%	Cooked sausages formulated with functional ingredients (CP or P fiber) showed attributes such as color, sweetness, astringency, bitterness, pork meat aroma, and a firm, pliable texture.	Díaz-Vela et al., 2017 [125]
Fiber	NR	Adding pomegranate peel flour (PPF) to chorizos resulted in a tougher texture, crumbly consistency, and less intense color.	2 and 4%	PPF is a functional ingredient that can replace some fat in raw meat products such as chorizo, enhancing texture, coloration, and shelf life through its polyphenols and dietary fiber, which lower water activity and promote a lower pH.	Maillard-Berdeja et al., 2022 [127]
Peptides from pork	Dipeptidyl peptidase IV (DPPV-IV) inhibitor	Pork meat		Bioactive peptides regulate type 2 diabetes by influencing enzymes in carbohydrates metabolism, insulin secretion, and incretin hormones such as GIP and GLP-1, thereby impacting postprandial blood glucose levels.	Martini et al., 2019 [135]
Peptides from slaughterhouse	Anserine and carnosine chelate copper; anserine’s stability in serum and resistance to degradation are due to methylation. Anserine is found in the skeletal muscle and brain of mammals and birds.	Chicken slaughterhouse by-products (CSBP)		CSBP hydrolysates show diverse health benefits, including antioxidant, antidiabetic, anti-inflammatory, and cardioprotective properties. Studies on antioxidant and antihypertensive effects suggest potential therapeutic applications research into their bioactive peptide content and health implications.	Ibarz-Blanch et al., 2023 [139]
Peptides as biomarkers	Anserine (β-alanyl-3 methylhistidine) is a dipeptide derived from carnosine, consisting of β-alanine and methylated 3-methylhistidine.	Anserine and carnosine are abundant in poultry, particularly in chicken and turkey.		Anserine (beta-alanyl-3-methyl-L-histidine) supplementation improved memory functions in AD-model mice by exerting a protective effect on the neurovascular units, which are composed of endothelial cells, pericytes, and supporting glial cells.	Creighton et al., 2022; Kaneko et al., 2017 [142,152]
Polyphenolics	Curcumin, carvacrol, thymol, cinnamaldehyde. ⤉	Curcumin or commercial microencapsulated phytogenic supplement	CU-with 50 mg/kg of curcumin	Curcumin with or without a phytogenic agent improved meat quality, with increased antioxidant levels and reduction of lipid peroxidation.	Galli et al., 2020 [115]
Polyphenolics	Phenolic acid, anthocyanins, and flavonoids, including monomeric phenolic compounds, such as (+)-catechins, (−)-epicatechin, and (−)-epicatechin-3-O-flattened dimeric, trimeric, and turmeric procyanidins. ⤉	Grape seed extract (GSE)	100 and 300 mg GSE/kg	Antioxidant enzymes of rabbits (superoxide dismutase, catalase, glutathione peroxidase, glutathione transferase) and total antioxidant capacity in blood were increased (*p* ≤ 0.05) by adding dietary GSE.	Hassan et al., 2016 [116]
Polyphenolics	Phenolic acid, anthocyanins, and flavonoids. ⤉	Wine-making by-product meal (WBM) contains concentrated phenolic compounds, as quantified in its crude extract.	0.5, 1 and 3%	In analysis of the WBM extract by HPLC, 25 phenolic compounds were observed, one of which was below the limit of quantification (procyanidin A2), totaling 9.51 mg phenolic compounds per gram of extract. The flavonol group was the major (36.4%), followed by anthocyanins and tannins with 24.07% and 13.5%, respectively.	de Alencar et al., 2022 [122]
Polyphenolics	NR	Plum puree, prunes (dried plum), and plum extracts.	3% plum extract	Treatment had a reduced (*p* < 0.05) TBARS value of 0.84 mg MDA/kg meat after 7 days of storage at 4 °C.	Ahmad et al., 2015 [123]
Probiotics	NR	*LAB: Lb. plantarum, Lb. paraplantarum, Lb. brevis, Lb. rhamnosus, Lb. sakei, Lb. zeae, Lb. paracasei, Ent. faecalis, Ent. faecium, Leuc. mesenteroides, Ped. pentosaceus, Ped. acidilactici, W. cibaria, W. viridescens, Lb. sake, Lb. curvatus,* and *Lb. plantarum*	NR	Dried fermented sausage (salami, salsiccia, soppressata, alheiras, botillo, chorizo, salchicón, pepperoni).	Martín et al., 2006; Tamang et al., 2016 [120,121]
Probiotics	NR	Analysis of auto-aggregation ability of LAB. Six thermotolerant lactic acid bacteria were isolated from cooked meat products (Vienna sausages)	62.6, 71.9, and 87.7%	*E. faecium* UAM1 showed significantly higher adherence (around 20%) to human Caco-2 cells compared to *P. pentosaceus* strains (2–5%) and *Lactobacillus acidophilus* LA-5 (6%). These findings suggest that *E. faecium* UAM1 has probiotic potential and may competitively colonize the intestinal tract.	Hernández-Alcántara et al., 2018 [128]
Digestive byproduct	Trimethylamine N-oxide (TMAO) is a small colorless amine oxide generated from choline, betaine, and carnitine by gut microbial metabolism.	Red meat, poultry, or fish.	NR	TMAO and its precursor choline in plasma predict cardiovascular disease risk in individuals undergoing cardiac evaluation. TMAO’s proatherogenic effects stem from the gut microbiota transforming phosphatidylcholine found in foods.	Wang et al., 2011 [153]
Tannins	Hydrolysable tannins (HTs) and condensed tannins (CTs). ⤉	Essential nutrient complex (ENC) extracted from chestnut wood (*Castanea sativa*)	0.5 and 1% ENC	ENC did not negatively affect carcass or meat traits in rabbits. It demonstrated antioxidant benefits at 0.5% inclusion but exhibited pro-oxidant effects at 1%. ENC had minimal impact on the fatty acid profile of rabbit meat.	Liu et al., 2009 [154]
Amino acids (AA) biomarkers	Urinary carnosine, 1-methylhistidine and 3-methylhistidine	-	1.5 g/kg per d	Urinary and plasma amino acids are potentially valuable biomarkers for assessing meat protein intake in diets primarily consisting of pork, beef, and chicken as main protein sources.	Altorf-van del kuil et al., 2013 [146]

⤉ = Metabolites with health benefits due to the effect of animal nutrition; NR = Not reported.

### 4.3. Eggs and Egg By-Products

Eggs from chicken are considered functional foods because they can deliver a high quality of protein, a balanced amino acid content, and a low calorie (75 kcal) input. Fats found in eggs (mainly in yolk) include saturated, monounsaturated, and polyunsaturated fatty acids; the latter are widely considered healthy, especially n-3 fatty acids. The main saturated acids are palmitic acid (C16:0) and stearic acid (C18:0), while the monounsaturated principal is oleic acid (C18:1 n-9). Finally, the polyunsaturated docosahexaenoic acid DHA (C22:6 n-3) is an essential compound for a correct brain development. Eggs also contain cholesterol, vitamins, minerals, and carotenoids such as lutein and zeaxanthin. Additionally, eggs are very versatile for cooking, a characteristic that is not easily found in other foods of animal or plant origin [154]. In terms of vitamins, eggs contain vitamins A, B, D, E, and K. Apart from calcium, eggs are rich in all minerals, especially iron and phosphorus. Research has been conducted to modify the composition of n-3 fatty acids and carotenoid content of eggs by modifying the diet of hens. In this era of innovation, it is crucial to appreciate the value of eggs and offer consumers a choice of “customized eggs” to potentially combat chronic diseases [155].

Since their high lipid content, egg yolks can be enriched by the inclusion of conventional or unconventional ingredients in the hen diet to boost compounds such as n-3 fatty acids. However, it is important to consider that increasing the content of fatty acids can make them more susceptible to oxidation, so it is desirable to fortify bird diets with antioxidants such as vitamin E, selenium, and carotenoids. Studies have focused on analyzing the properties of fruits, vegetables, grains, and herbs and their potential role in preventing degenerative diseases. Yet, there is research on the antioxidant content found in animal products. Particularly, vitamins A and E, along with selenium and carotenoids, possess antioxidant qualities that can be enhanced by adjusting the bird’s diet [156].

However, endogenous antioxidants in animal-derived products are often lost during processing, handling, or storage, requiring additional supplementation with exogenous antioxidants. In practice, the addition of antioxidants during processing plays a significant role, as the compounds added have the potential to enhance the activity of inherent antioxidant systems, inhibit product degradation, and delay the appearance of unpleasant flavors and colors, thereby increasing shelf life. Therefore, natural antioxidants are distributed, retained, and remain functional in animal-derived products, but there is an issue with this supplementation as their bioavailability may be low to observe their benefits. That is, despite a high concentration in diet, blood levels may be low, and their concentration in tissues, such as liver, muscle, and egg yolk, among others, is often not significant [6]. For this reason, research projects aimed at increasing total lipids, n-3 fatty acids, and pigments through the manipulation of ingredients in the birds’ diet, including fish meal and oil, crustacean meal, seaweed, acid oils, selenium, and copper, among others, have emerged. It should be noted that by increasing the lipid portion, the possibility of oxidation of these fatty acids increases, so the aforementioned unconventional sources also play an important role as antioxidants. An antioxidant can be defined as “any substance that delays, prevents, or eliminates oxidative damage to a ‘purpose’ molecule” [157], or “any substance that directly eliminates reactive oxygen species (ROS) or that acts indirectly to increase antioxidant defenses or inhibit ROS production” [158]. In this regard, most research has been carried out using eggs as a medium to enrich diet with n-3 fatty acids and antioxidants. It has been demonstrated that marine-derived EPA and DHA fatty acids have been shown to be effective in the treatment and prevention of cardiovascular, neurodegenerative, and rheumatoid arthritis diseases because they participate in modulating the immune response. Several clinical and epidemiological studies mention that the consumption of EPA and DHA may contribute to the prevention or treatment of various pathologies, especially those where inflammation plays a significant role in their development [159]. On the other hand, the use of certain carotenoids as additives in poultry feed is aimed at improving the color of egg yolks. Additionally, they help neutralize singlet oxygen and free radicals and protect against oxidative damage [156]. Sardine oil, langostilla (red shrimp) flour, conjugated linoleic acid, tuna oil, shrimp meal, squid flour, and selenium have been used for this purpose. Langostilla meal (LM) is made from those crustaceans that do not meet the market parameters (size and weight) of the standards for sale and/or export, so they are processed to obtain meal and are used in animal feed devoted to shrimp and fish. This meal is an excellent source of protein (36%), ether extract (0.9%), and astaxanthin (1.4 mg/100 g), which is responsible for the pink color of shrimp and other crustaceans and flamingos, and it is considered a better antioxidant than vitamin E [160]. LM has been studied from different perspectives in the feeding of laying hens in order to modify, in the egg, the lipid fraction and yolk pigmentation. Carranco et al. [161] conclude that the inclusion of up to 20% LM showed a slight increase in fatty acids and yolk color, and that while as the storage time occurred, these results decreased, especially after 30 days/20 °C, so it is suggested that the egg modified with LM be consumed before 15 days of being laid [162,163].

The crustacean known as the red crab, or shrimp mantis (*Pleuroncodes planipes*), an abundant resource along the coast and Gulf of Baja California, Mexico, has also been studied as an alternative in the feeding of laying of hens due to its high protein content (33.7%), extract ether (7.3%), and astaxanthin (10.9 mg/100 g) [164]. In several studies conducted on this crustacean [165,166,167], it has been reported that the albumen fraction underwent little modification, i.e., a small increase in protein content. However, in the egg yolk, changes were observed in total lipids, fatty acid profile, and pigmentation. Carrillo-Domínguez et al. [167] and Calvo et al. [165] used shrimp mantis flour at inclusion rates of 3%, 6%, and 9% in laying hen diets to evaluate the effects on cholesterol concentration and the content of n-3 and n-6 fatty acids. The results and conclusions reported that total lipids, fatty acid profile, EPA, DHA, ALA, and AA increased compared to the control. Therefore, up to 6% HL can be included in feed for layer hens, providing good yolk coloration. Calvo et al. [165] and Carranco et al. [166] worked with this same crustacean at inclusion rates of 4% and 6%, respectively, in laying hen diets, with the aim of enriching the egg with fatty acids and astaxanthin as a red pigment and antioxidant, as well as analyzing lipid peroxidation during 15 and 30 days of storage at between 4° and 20 °C. They obtained an increase in the fatty acid profile. However, at 30 days of storage at both temperatures, the presence of reactive species to thiobarbituric acid (TBAR’s) was detected, as well as a rancid and humid smell in the egg, concluding that 4% and 6% of HL increase the content of fatty acids, achieving good yolk pigmentation, and recommending that to avoid oxidation, the product should be consumed before 15 days of storage. In another study, black tuna flour was used at inclusion rates of 1%, 2%, and 3% in laying hen diets to increase the content of eicosapentaenoic acid (EPA) and docosapentaenoic acid (DHA). The results reported that as the level of inclusion of the black tuna flour increased, the levels of cholesterol increased, and total lipids were not different between inclusions. However, the DHA content increased relative to the inclusion percentage of black tuna flour. Additionally, a decrease in n-6 fatty acids, linoleic and arachidonic acid, was observed [168].

While seeking new feeding strategies for birds, research was conducted on the utilization of various seaweed species: *Sargassum sinicola,* which is abundant in the Pacific Ocean (Gulf of California), Gulf of Mexico, and Caribbean waters. This resource is low in energy and protein but rich in minerals. *Macrocystis pyrifera* (Giant kelp) grows on rocky substrates in sargasso forests. *Ulva lactuca* has a wide distribution along both the Pacific and Atlantic coasts, with several countries using it for animal and human consumption, and it is used in the gastronomic industry and as a dietary supplement for birds, sheep, and cattle, with high ash and carbohydrate content. These marine resources contain natural chemical compounds such as n-3 fatty acids, sterols, and complex carbohydrates. The latter are mainly nondigestible carbohydrates such as alginic acid, laminarin, mannitol, cellulose, and fucoidan, which are the focal components of the cell wall. Seaweed contains sterols in their unsaponifiable lipid fraction, such as sitosterol, fucosterol, chondrasterol, and ergosterol. Brown seaweeds contain additional 24-methylene-cholesterol, fucosterol, and saringosterol; and green seaweeds present beta-sitosterol, 24-methylene-cholesterol, and 28-isofucosterol. These sterols have the ability to reduce blood cholesterol concentrations, and there is a trend towards reducing fat accumulation in the liver and heart [169,170].

Given the rich algae resources in the Baja California Peninsula, Mexico, these have been studied to understand their chemical composition as they are potential components for human and animal diets. The algae studied were classified into green, red, and brown. With green algae, the following species were evaluated: *Ulva* spp., *Enteromorpha intestinalis*, *Caulerpa sertularoides*, and *Bryopsis hypnoides*. Under clasification h red algae: *Laurencia johnstonii*, *Spyridia filamentosa*, and *Hypnea valentiae*. Lastly, with brown algae: *Sargassum lierporizum*, *Sargassum sinicola*, *Padina durvillaei*, *Hydroclathrus clathratlius*, and *Colpomenia sinuosa*. These algae were collected, dried in the sun, and subsequently ground. The results showed that the protein content in the algae was less than 11%, except for *L. johnstonii* with 18% and low energy content. The content of extractive ether was generally lower than 1%, with the most abundant fractions being total carbohydrates and inorganic matter, which are the main components of these resources [169,171].

This research included the incorporation of marine algae *Sargassum* spp. at levels (2%, 4%, 6%, and 8%) into the diets of laying hens to assess its impact on egg yolk cholesterol content. The findings revealed a decrease in cholesterol concentrations (mg/100 g fresh egg), with higher inclusion levels of *Sargassum* spp. [171,172]. Likewise, soybean oil by-products have been used to replace crude soybean oil, allowing for no competition in human food applications. Soybean oil by-products are one form of the vegetable oil refining process, are more economical, and have a chemical composition consisting of free fatty acids (59%), phospholipids, non-saponifiable ingredients, oxidation compounds, carotenoids, and xanthophylls. Pérez et al. [172] conducted a study with egg-laying hens, including soybean oil by-products in substitution of crude soybean oil. The authors concluded that the addition of anticoccidial compounds in layer hen formulations results in the deposition of certain fatty acids in different proportions. Though these inclusions did not affect the productive variables, they observed an improvement of Haugh units and a reduction in the production cost of the egg.

Another essay on productive variables, egg quality, bioactive compounds, and bird immunity was conducted by Ahmad et al. [173]. This time, the effect of including *Moringa oleifera* (Lam.) pod flour was evaluated. Four diets were formulated: A (control) and *Moringa* at inclusions of 0.5%, 1.0%, and 1.5%. The results showed that feed conversion ratio and egg mass decreased, with the lowest values at 0.1%. However, β-carotene, quercetin, and selenium levels in the yolk increased, while cholesterol concentration decreased compared to the control diet. Proximate analysis of the yolk revealed that crude protein, ash, potassium, calcium, magnesium, and phosphorus increased with *Moringa* inclusions, while ether extract data decreased compared to the control. Serum biochemical indicators, including glutamic-pyruvic transaminase, glucose, creatinine, and cholesterol, significantly decreased with *Moringa* incorporation. The conclusion of this study indicated that *Moringa* has positive effects on productive variables and bird immune status, with the 1.5% inclusion showing the best results. Additionally, *Moringa* could be an alternative to promoting growth, antioxidant content, and performance of laying hens.

Other resources such as carrots (*Daucus carota* L.) and beets (*Beta vulgaris* L.) contain the pigments β-carotene and betalain, respectively, as well as antioxidants and provitamin A, and can be used as meals to feed laying hens. De Souza et al. [174] worked with these two tuber species; both species were lyophilized and arranged into five experimental diets to evaluate productive parameters, egg quality, and retinol concentration in the eggs. The formulations were: T1 (corn and soy), T2 (sorghum and soy); these two were the controls; T3 (sorghum and carrot at 0.8%), T4 (sorghum and beet at 0.8%), and T5 (sorghum with 0.4% carrot and 0.4% beet). The trial with the birds lasted 63 days, and the results showed no differences (*p* < 0.05) in productive variables among the five treatments. Adding carrot or beet flour in sorghum-based diets at the concentration of this study was not sufficient to reach a retinol concentration similar to the corn and soybean meal diet. However, the authors concluded that using 0.8% carrot and beet flour increases retinol concentrations and yolk color compared to the sorghum-only diet, but the values were lower compared to the corn diet.

Ortíz et al. [175] tested various essential oils from rosemary, thyme, and oregano, which were incorporated to replace antioxidant additives such as BHT (butylated hydroxytoluene) and BHA (butylated hydroxyanisole) in bird diets. Oregano essential oil (*Lippia origanoides* Kunth) (OEO) was added to the bird feed to evaluate its effect on productive variables, lipid profile, and oxidative stability (TBAR’s) in eggs enriched with polyunsaturated fatty acids (PUFAs), analyzed fresh and stored for 30 and 60 days at 4 °C. Two formulations were prepared: T1 palm oil (PO) with OEO and T2 fish oil (FO) with OEO, for an eight-week trial. Among the results reported by these authors, it is highlighted that productive variables were not affected (*p* > 0.05). Regarding the concentration of polyunsaturated fatty acids, there was a 16.8% increase in the egg with FO and OEO, with a 1.4% increase in DHA. In terms of oxidative activity, the results expressed in malondialdehyde (MDA) concentration increased in the egg and improved oxidative stability during storage. The fatty acid profile increased with FO inclusion in PUFA and DHA content, which in turn favored MDA concentration in the egg and during storage. As expected, lipid oxidation increased, being higher at 60 days. The authors concluded that enriching eggs with PUFAs by including FO is a suitable alternative. Furthermore, OEO can be used as a replacement for synthetic antioxidants, as it showed stability for up to 60 days of egg storage at 4 °C. However, they recommend further studies with higher EOE inclusion percentages.

Carranco-Jáuregui and colleagues [176] conducted research using *Tithonia diversifolia* leaf meal (TDM) by increasing the levels of lutein and zeaxanthin in the diets of laying hens. Their aim was to boost the carotenoid content in eggs and enhance their yolk coloration. For the study, five experimental groups were arranged as follows: T1 control diet and 15 ppm yellow pigment; T2 control diet supplemented with 1.8% TDM and 15 ppm of xanthophylls; T3 control diet together with 5% TDM and 42.5 ppm of xanthophylls; T4 control diet supplemented with 10% TDM and 85 ppm of xanthophylls; and to conclude, T5 control diet and 15% TDM and 127.5 ppm of xanthophylls. Red pigment was not included in the bird diets during the three weeks of the study, but it was added in the subsequent three weeks. The yolk was quantified for total carotenoids, lutein, zeaxanthin, and capsanthin. The results indicated that TDM can be considered as a good alternative for poultry feeding up to a 10% inclusion rate. At this percentage and in combination with red pigment (canthaxanthin), orange yolks and a natural source of carotenoids were guaranteed.

In the same line, Botsoglou et al. [177] conducted a study to evaluate the impact of adding saffron stigma meal (RSSM) versus α-tocopherol into hen diets to observe the oxidative stability changes of eggs. The diets were as follows: (1) control diet, (2) control diet plus 10 mg/kg RSSM, (3) control diet plus 20 mg/kg RSSM, and (4) control diet plus 200 mg/kg of α-tocopherol. After six weeks of experimentation, eggs from hens were collected, and the lipid oxidation was evaluated. Such a test was performed on refrigerated whole eggs and yolks at room temperature, adjusting the pH to 6.2 and 4.2. The results showed that the degree of lipid oxidation in whole eggs measured by malondialdehyde (MDA) formation (ng/g) was different between treatments but did not change over time. Yolks adjusted to a pH of 6.2 yielded high MDA values (ng/g) for group 2 and higher than group 3, indicating that RSSM had antioxidant activity depending on the inclusion level. Thus, eggs from the CON group presented higher (*p* < 0.05) MDA values than those of all other groups, whereas eggs from the TOC group had lower (*p* < 0.05) MDA values than all other groups. The SAF group presented lower (*p* < 0.05) MDA values than the ORE and ROS groups.

In another study, laying hen diets were supplemented with tomato powder at 5 and 10 g/kg. Performance, egg quality, serum carotenoids, vitamins, and malondialdehyde (MDA) concentration were measured. The results showed a linear growth in performance and egg quality but a linear decrease in feed conversion. The serum lycopene and yolk egg yolk β-carotene, lutein, and vitamin A increased in the two diets with tomato powder, while MDA and lipid peroxidation decreased with the increase in tomato powder in the diets [178]. Kara et al. [179] studied the supplementation of raisin pomace in corn and soy-based laying hen diets. The inclusion of raisin pomace in the diets was 4% and 6%. The results showed no significant difference in productive parameters, physical egg quality, total cholesterol, total proteins, and triglycerides compared to the control group. However, plasma and egg yolk MDA and serum glucose levels decreased significantly, as well as in the egg yolk. The authors concluded that raisin pomace supplementation has the potential to extend the shelf life of eggs.

Imbaquingo [180] studied the use of pigweed leaf meal (*Amaranthus retroflexus*) at 5%, 10%, and 15% in quail diets to assess its impact on productivity, egg quality, and economic performance. Results showed no significant differences in food consumption, egg weight, shape index, shell thickness, yolk, and albumen percentage. However, the 10% inclusion led to higher egg production and feed conversion. These same results also transfer into richer yolk color and increased protein concentration. The study concluded that incorporating 10% pigweed leaf flour improved egg production efficiency and cost-benefit ratio, while 15% enhanced internal egg quality, making it a promising alternative feed for laying quails. Research on ginger’s anti-inflammatory properties in humans led to a study using ginger flour (*Zingiber officinale*) in quail diets. The study evaluated productivity variables and egg quality with different ginger flour levels (T1: 0%, T2: 0.2%, T3: 0.4%, T4: 0.6%) over five laying cycles. Results indicated balanced food consumption with ginger inclusions, favoring palatability and nutrient utilization. The 0.4% inclusion showed the best results compared to the control, particularly in egg quality improvement and food consumption in later stages [181]. In another study, feeding olive cake to laying hens has been found to impact yolk lipid composition. Tannin supplementation to lactating dairy cows hampered the oxidative stress, while increasing the anti-inflammatory cytokines (IL-10, IL-8, IL-1β) during winter and summer feeding with contrasting forage qualities [10].

Degollado [182] investigated the effects of moringa meal (*Moringa oleifera* Lam.) in quail diets on laying percentage, energy utilization, metabolizable protein, and egg quality. Three diets (T1: 0%, T2: 5%, T3: 10%) replaced sorghum and soybeans. The study spanned 8 weeks, with a 2-week food adaptation period. While no significant differences were observed in food consumption, weight gain, egg production, yolk, albumen proportion, energy, and metabolizable protein utilization, the *Moringa* meal inclusion at 10% positively impacted egg weight, shell thickness, shape index, and shell proportion. Color values were higher in T2 and T3 compared to T1. Adding *Moringa* meal up to 10% at the start of the laying cycle showed positive effects on the studied variables.

Several studies have demonstrated that polyunsaturated n-3 fatty acids (EPA and DHA) are effective in preventing cardiovascular, degenerative disease, cancer, and inflammatory conditions, among others. Therefore, research is being conducted to promote the consumption of these PUFA to incorporate them into the diet. In this regard, eggs are a widely accepted food with high nutritional value. They can be positively modified through strategic feeding of birds, thus achieving what are known as value-added or functional eggs [163,176,183]. In summary, the Table 4, showed metabolites and health benefits and implications of avian eggs.

### 4.4. Fish and Fish By-Products

Omega-3 fatty acids, which are commonly found in marine products such as tuna, mackerel, and salmon, are essential for maintaining good health in humans. Specific fatty acids, such as eicosapentaenoic acid (EPA) and docosahexaenoic acid (DHA), quickly become part of cell membranes. Both fatty acids possess powerful anti-inflammatory properties. EPA and DHA are important for preventing and treating health issues that stem from inflammation. They are precursors of substances such as resolvins that reduce inflammation and block agents that promote inflammation. showing benefits in conditions, including heart disease, brain disorders, arthritis, and injuries caused by restricted blood flow [159].

Marine fats contain omega-3 polyunsaturated fatty acids, with α-linolenic acid playing a crucial role in producing EPA and DHA. These fats are important for growth, development, and metabolic processes, lowering cholesterol and triglyceride levels and assisting in the management of conditions such as low blood pressure, arthritis, autoimmune disorders, and cancer. Fish oils have undergone processing to amplify their impact and shelf life for consumption, underscoring their significance in maintaining health and treating illnesses [187]. Omega-3 fatty acids have proven to be highly effective in the management and prevention of illnesses. Research studies have backed up their heart benefits in individuals with type II diabetes, reducing the chances of heart related problems, decreasing levels, and boosting high density lipoprotein levels. These essential fatty acids also play a role in the treatment of cancer, asthma, psoriasis, Crohn’s disease, multiple sclerosis, migraines, arthritis, kidney issues, mental health conditions, and depression, as well as, in prenatal and postnatal development by supporting brain cell function and growth [188].

Schwartz et al. [189] conducted a review aimed at evaluating how safe vitamin A and fish oils docosahexaenoic acid (DHA) are in slowing down the advancement of retinitis pigmentosa (RP). RP is an eye condition marked by the deterioration of retinal photoreceptors, leading to significant vision impairment and potential blindness. Common symptoms include difficulty seeing at night and loss of vision, eventually impacting vision. With a prevalence rate of 1 in 4000 individuals in the USA, there is currently no established cure for RP. These studies sought to determine whether vitamin A, fish oils, or a combination of both could potentially delay the progression of vision loss in individuals with RP. This study research comprised four trials involving 944 participants aged 4 to 55 years. These trials evaluated the impact of DHA alone, vitamin A alone, and a combination of DHA and vitamin A versus vitamin A alone on visual acuity over a follow-up period of 4 to 6 years. The study focusing on DHA did not demonstrate a significant effect on visual acuity in the 41 participants. The authors concluded that based on the results of the four studies, there is uncertainty regarding the efficacy of treatment with vitamin A, DHA, or both for individuals with RP. The review highlighted the lack of clarity on whether vitamin A, fish oils, or their combination have a discernible impact on delaying the progression of vision loss in individuals with RP. Further research is warranted to elucidate the potential benefits of these interventions in managing RP.

Marine sources of n-3 polyunsaturated fatty acids (PUFAs) have been extensively researched for their ability to protect the brain in animal models of stroke, resulting in improved outcomes [190]. Stroke is associated with a major cause of disability and often requires long-term specialized care for patients. A transient ischemic attack (TIA), also commonly known as a “mini stroke”, involves a temporal disruption of blood flow to brain circulation. Thus, the n-3 fatty acids eicosapentaenoic acid (EPA) and docosahexaenoic acid (DHA) found in oily fish play crucial roles in brain function. Studies on animals indicate that EPA and DHA can safeguard brain cells following a stroke, particularly when given after the incident. As well, numerous studies have extensively examined the benefits of consuming polyunsaturated fatty acids (PUFAs) for preventing certain diseases. A Cochrane review suggests that increasing PUFA intake may slightly lower the risk of heart disease and cardiovascular events. The review analyzed 49 randomized controlled trials involving 24,272 participants who either increased their PUFA intake or maintained their diet. The initial PUFA intake ranged from 3.9% to 8% of energy intake across trials [191]. These findings indicate that boosting PUFA consumption may not significantly impact mortality but could reduce heart disease and cardiovascular events. It might also lower the risk of death from heart disease and stroke, though its effect on mortality remains inconclusive due to limited quality evidence. The impact of PUFAs on cardiac cerebrovascular events and atrial fibrillation is uncertain given the very low quality of available evidence. When it comes to lipid profiles, increasing PUFA intake may slightly lower triglycerides, with an impact on cholesterol and LDL or HDL levels [191]. Moreover, boosting PUFA intake is unlikely to have an effect on body fat. The effects of PUFA consumption on health issues such as pulmonary embolism and bleeding are uncertain due to the low quality of evidence available. A study in the British Journal of Nutrition indicates that n-6 PUFA intake can reduce LDL cholesterol levels in the blood, without showing a link to obesity in humans. The authors suggest an intake of n-6 PUFA exceeding 5%. Around 10% of energy based on existing data supports this recommendation [192]. Combining this with a reduction in SFA intake could enhance the benefits.

Despite these findings, a Cochrane Database of Systematic Reviews published a study in 2020 that examined the effects of marine-derived n-3 PUFAs on functional outcomes and dependency in stroke patients. Thirty randomized controlled trials were identified, with nine presenting outcome data and a total of 3339 participants. The dose of marine-derived n-3 PUFAs varied from 400 mg/day to 3300 mg/day. The review found low certainty evidence for short-term follow-up (up to three months) and no evidence for longer-term (more than three months) follow-up of an effect of the intervention on mood, other types of stroke, or quality of life [190].

In the same line, a recent review of 14 randomized trials involving over 20,000 patients who had heart issues looked into the impact of EPA and DHA in preventing heart problems [193]. The results did not show proof that omega-3 fatty acid supplements could significantly lower the risk of heart disease. A trial in a community group revealed that higher levels of LA were linked to chances of dying from heart disease, while AA levels did not seem to affect heart health. The research on the effects of PUFAs on health is conflicting. Fish oils contain omega-3 PUFAs, and plant oils have omega-6 PUFAs. It seems that consuming food or supplements rich in PUFAs could help lower cholesterol levels but might also lead to weight gain, making it unclear how they exactly impact health. Therefore, more studies are necessary to determine the amount of PUFAs for maintaining heart health.

Research has been conducted on the benefits of omega-3 fatty acids often found in fish oil for maintaining permeability in individuals with end-stage renal disease undergoing hemodialysis. The rationale behind this is that omega-3 fatty acids could help to reduce the risk of blood vessel blockages, which are issues for heart disease patients relying on effective blood circulation during hemodialysis. A review published in the Cochrane Database of Systematic Reviews in 2018 looked into the use of omega-3 fatty acid supplements versus a placebo in no treatment to maintain patients undergoing hemodialysis [194]. This review included five randomized controlled trials involving a total of 833 participants, one of which was a pilot study with 7 of them. While four studies focused on patients with grafts, one study involved arteriovenous fistulas. It is necessary to wait 6 to 12 months to obtain results. The study findings suggest that there is evidence indicating that fish oil supplements do not prevent blood vessel blockages or cause any harm in arteriovenous fistula patients. Nevertheless, this data is based on one research study. For patients with grafts, there remains uncertainty surrounding the prevention of blockages or the likelihood of experiencing serious complications. However, there is a possibility of encountering issues such as bloating, gas, or an unusual taste in the mouth.

Overall, the authors conclude that there is limited high-quality data on the beneficial effects of omega-3 fatty acid supplementation for preventing HD access occlusion in patients with renal insufficiency. While there is no solid evidence to suggest that fish oil with omega-3 can prevent vascular access occlusion during HD or increase the risk of severe or non-severe side effects, all evidence of occlusion prevention comes from one or two studies, necessitating more and higher-quality studies to establish the potential benefits of omega-3 fatty acid supplementation in this population. Table 5 showed some metabolites of fish, health benefits, and implications.

## 5. Conclusions

Bioactive compounds in animal diets, either from grazing environments or by means of pre-formulated diets, have shown promise in boosting the bioactive properties of these products and potentially impacting chronic disease in both animals and humans. However, there are still gaps in our knowledge, particularly related to tracking these metabolites from animal consumption to their presence in humans, as well as in animal products. To progress in this field, it is crucial to study the variety of feedstuffs consumed by animals and to understand how these bioactive compounds change within an animal’s biology for maximizing the presence in animal-based foods and ultimately enhancing their health benefits. Additionally, diversifying the plant species intake by animals not only supports sustainable livestock farming, but also boosts the nutritional and bioactive content of resulting animal products. These approaches are established to enhance the value of animal products and contribute to sustainable livestock farming practices. However, there is still much to explore in this domain. Key research inquiries seem to point the same way in regarding how different plant byproducts affect the nutritional content and sensory characteristics of animal-derived goods. It is essential to determine the dosages and application methods for these bioactive substances in animal diets.

Furthermore, it is vital to comprehend how these plant-derived compounds interact with feed components within an animal’s digestive system. Additionally, evaluating the long term effects of feeding animals with industrial residues is critically important. This becomes especially relevant due to the chemical compositions and potential presence of antinutritional elements in these by-products, along with challenges related to feed regulations. Bringing these knowledge gaps together will not just boost our understanding of animal nutrition but also carry important implications for human wellbeing. It is vital to investigate the metabolic pathways and bioactivity of plant secondary metabolites in animals, especially across various physiological conditions. This insight will aid in the use of agroindustrial by-products in animal feeding, overcoming challenges such as chemical variations and regulatory limitations.

Additionally, there is a need for research concentrating on the effects and effectiveness of these plant-based compounds in animal products post-consumption. The potential impact of plant metabolites on the nutritional value and health-promoting attributes of animal products remains largely unexplored, particularly regarding their role in managing chronic diseases in humans. Exploring this field could provide insight into creating functional foods from animal sources, which could significantly contribute to preventive healthcare strategies. Therefore, incorporating plant bioactive elements into animal diets presents an opportunity to enhance the nutritional quality of food derived from animals. This method not only supports sustainable livestock farming but also holds promise for improving public health outcomes. By conducting research to connect existing knowledge gaps, we can tap into the complete capabilities of plant secondary metabolites in animal nutrition. This comprehensive strategy holds the promise of enhancing the wellbeing of both animals and humans, paving the way for bioactive compounds from plant-based diets to play a role in contemporary animal farming methods. As a perspective of the present review, it offers a starting spot to pay attention to the undeniable urgent need of sustainable animal-origin food production.

## Figures and Tables

**Table 1:** Animal feedstuffs classification of the National Research Council [23].

NRC Feed Classification	Description	Main Characteristic	Example of Feedstuff	Main Metabolites	Author
1	Dry forages and roughages	≥18% fiber	Browse/range plants	Tannins and Saponins	Okunade et al., 2014 [24]
2	Pasture, range plants and forages fed fresh	≥18% fiber	Sainfoin Trifolium	Tannins Isoflavones	Mueller-Harvey et al., 2019 [20] Hloucalova, et al., 2016 [25]
3	Silages	≥18% fiber			
4	Energetic sources	≤20% protein and ≤18% fiber	Citrus pulp Corn	Hesperidin, narangin Anthocyanins	Simitzis, and Deligeorgis, 2018 [6] Antunović et al., 2022 [26]
5	Protein sources	≥20% protein and ≤18% fiber	Alfalfa Clover	Flavonoids Isoflavones	Dabbou et al., 2018 [27] Dadáková et al., 2020 [28]
6	Minerals	Guaranteed analysis	Selenium	Selenites with tetravalent (Se4+), and Selenates with hexavalent (Se6+) cations	Pecoraro, et al., 2022 [29] Gu and Gao, 2022 [30]
7	Vitamins	Guaranteed analysis	Retinyl acetate	Vitamin A	Shask and Pelletier, 2023 [31]
8	Additives	Specific	Prebiotics	Inulin	Pandey et al., 2019 [32] Juárez et al., 2019 [33]

**Table 4:** Metabolites and health benefits and implications of avian eggs.

Metabolite Category	Metabolite	By-Product Added to Feedstuff	Dose, Concentration, or Treatment	Biological Function of Metabolites, Biochemistry, and Biotransformation	Reference
Polyunsaturated fatty acids and astaxanthin	EPA, DHA, ALA and AA, and astaxanthin	Red crab meal	3%, 6%, and 9%	The results were that total lipids, EPA, DHA, ALA EPA, DHA, ALA, and AA fatty acids increased with respect to the control.	Carrillo et al., 2005 [167]
Polyunsaturated fatty acids and astaxanthin	Polyunsaturated fatty acids and antioxidant	Red crab meal	4% and 6%	Inclusions of 4% and 6% red crab meal in diets for laying hens resulted in an increased fatty acid profile and egg yolk pigmentation.	Calvo et al., 2016 [165]
Polyunsaturated fatty acids and astaxanthin	Polyunsaturated fatty acids and antioxidant	Red crab meal	4%	The inclusion of 4% lobster meal in diets for laying hens allows obtaining eggs enriched with n-3 fatty acids and astaxanthin. Also, astaxanthin has an antioxidant function, protecting fatty acids.	Carranco et al., 2016 [166]
Pigments	Astaxanthin	Shrimp meal	4–25%	The results of this work showed that the color of the yolk was lower when compared to the control and decreased as the storage time passed (30 days/20° and 4 °C).	Carranco et al., 2003, 2006, 2011 [160,161,162]
Polyunsaturated fatty acids and astaxanthin	Polyunsaturated fatty acids and antioxidant	Shrimp meal	20%	This study evaluated the concentration of astaxanthin, fatty acids, and peroxidation of egg yolk stored for 15 and 30 days at room temperature and refrigeration. The differences observed were due to the normal deterioration that all perishable foods undergo during normal deterioration and prolonged storage.	Carranco et al., 2018 [163]
Carotene	Lutein, canthaxanthin	*Tithonia diversifolia* meal	1.8, 5, 10, and 15%	Leaf meal with petioles of *Tithonia diversifolia* can be considered as an alternative for poultry feed up to a level of 10% without affecting productive parameters and providing pigmentation to egg yolk.	Carranco et al., 2020 [176]
Protein	Protein	Giant squid meal	10 and 20%	This meal can be used in laying hen diets in no more than 10% so as not to affect production parameters and egg flavor. It also showed a slight increase in protein content.	Carranco et al., 2020 [183]
Polyunsaturated fatty acids and cholesterol	DHA, ALA, AA, LA, cholesterol	Black tuna meal (BTM)	1, 2, and 3%	Black tuna meal (3%) can be used to increase the fatty acids in eggs (DHA, ALA).	Rodríguez-Michel et al., 2018 [168]
Cholesterol	Cholesterol	*Sargassum* spp. algae	2, 4, 6, and 8%	*Sargassum* spp. was used in diets for laying hens, resulting in a significant decrease in egg cholesterol concentration.	Carrillo et al., 2012 [184]
Myristic and palmitic acid	Myristic and palmitic acid	Acidulated soybean oil	2 and 4%	Myristic and palmitic fatty acid concentration increased in concentration without affecting the value of stearic acid in eggs.	Pérez et al., 2019 [172]
Chitin, protein, amino acids, astaxanthin	Chitin, protein, amino acids, astaxanthin	Red crab meal	3, 6, and 9%	Astaxanthin contributes to pigment egg yolk.	Carrillo, 1993 [164]
Monounsaturated fatty acids Polyunsaturated fatty acids	Fatty acids and antioxidants	Oregano oil, palm oil, and fish oil	Oregano oil (100 g/ton) + palm oil (2%) Oil of oregano (100 g/ton) + fish oil (2%)	Oregano oil in the feed of laying hens is a natural alternative to increase PUFA fatty acids and to replace synthetic antioxidants used in the feed industry.	Ortíz et al., 2017 [175]
Carotene, vitamins and minerals	Lutein, vitamin E, selenium, zeaxanthin, and iodine	NR	NR	The use of certain carotenoids as poultry feed additives improves the color of egg yolk. They also help neutralize singlet oxygen and free radicals and protect against oxidative damage. Lutein and zeaxanthin present in the egg are also found in human serum, skin, and the ocular macula and will play protective roles against oxidative stress.	Aparicio et al., 2018 Nimalarante and Wu, 2015 [154,156]
Carotene	β-carotene, betalain	Freeze-dried carrot and sorghum Freeze-dried beet and sorghum Carrot, beet, and sorghum	0.4 and 0.8%	The use of 0.8% carrot and beet meal increased the retinol concentration and egg yolk color compared to the corn and soybean diets.	Souza et al., 2019 [174]
Carotene, quercetin, mineral, and serum biochemical markers	β-carotene, quercetin, selenium, and serum biochemical markers	*Moringa* meal Oleifera (Lam.)	0.5, 1.0 and 1.5%	*Moringa oleifera* pods could be used as alternative growth promoters, which improve antioxidant activity and the performance of laying birds.	Ahmad et al., 2017 [173]
Antioxidants	NR	Rosemary, oregano, saffron, and α-tocopheryl acetate	Basal an additional 200 mg α-tocopheryl acetate/kg, or rosemary at 5 g/kg diet, oregano at 5 g/kg diet, or saffron at 20 mg/kg diet.	Considering that egg yolks from the dietary supplemented groups exhibited increased resistance to lipid oxidation compared to control, one could say that antioxidant constituents of rosemary, oregano, and saffron passed from the feed into the developing yolk, providing eggs with increased antioxidant properties.	Botsoglou et al., 2005 [177]
Carotenes and vitamins	Lycopene, beta-carotene, lutein, vitamin A	Tomato powder	5 and 10 g/kg	Concentrations of lycopene in serum and egg yolk beta-carotene, lutein, and vitamin A increased powder, while MDA decreased linearly at both concentrations of tomato with increasing tomato powder. Tomato powder supplementation increased the concentration of carotenoids and vitamin A and a reduction of peroxidation.	Akdemir et al., 2012 [178]
Antioxidants	Serum cholesterol, total protein, glucose, triglycerides, and MDA	Raisin pomace	4 and 6%	The addition of raisin pomace significantly decreased plasma levels of MDA and serum glucose. Egg yield, egg quality, and serum levels of total cholesterol, total protein, and triglycerides were not negatively affected. Plasma and yolk MDA and serum glucose levels were reduced by 4% and 6% supplementation. By raising pomace, supplementation has the potential to extend shelf life.	Kara et al., 2016 [179]
Fatty acids	Pentadecanoic acid	*Azolla anabaena*	5, 10, and 15%	The inclusion of 5% Azolla showed better results on productive behavior, voluntary consumption, and apparent nutrient digestibility with respect to the control diet.	Buenaño, 2016 [185]
Fructooligosaccharides	Inulin	Inulin	Control diet with Ca without inulin (1) Diet with Ca and inulin (2) Low Ca diet with inulin (3) Low Ca diet without inulin (4)	Incorporating inulin in the feed had an effect on the shape index, and the yolk diameter was lower with the incorporation of inulin in the feed yolk diameter with diet 4.	Coronado, 2022 [186]
Antioxidants	productive parameters and egg quality	Ginger flour (*Zingiber officinale*)	0.2, 0.4, and 0.6%	The inclusion of ginger flour improves the productive parameters and the quality of the eggs.	Núñez et al., 2021 [181]

NR = Not reported.

**Table 5:** Metabolites of fish, health benefits, and implications.

Category	Metabolite	Feedstuff	Dose, Concentration or Treatment	Function	Reference
Hydrolyzed fish	Low-molecular-weight peptides	Fish food	60% protein, less than 5% lipids, and less than 10% moisture.	Hydrolyzed proteins encompass essential and non-essential amino acids, with notable levels of aspartic and glutamic acid derived from muscle, head, skin, and viscera. These hydrolyzates contain a significant proportion of peptides ranging from 500 to 2500 Da, followed by 200 to 500 Da. Fish exhibit heightened dipeptide and tripeptide absorption instead of free amino acids.	Cardoza-Ramírez, 2021 [195]
Hydrolyzed fish	Free amino acids and nucleotides	Fish food		Hydrolyzed fish contain free amino acids, such as glutamic acid, aspartic acid, glycine, arginine, alanine, proline, leucine, and isoleucine, along with specific nucleotides, which impart an attractive aroma to the feed, appealing to fish and shrimp. The inclusion of lysine, methionine, nucleotides, anserine, and taurine is proposed to elicit the secretion of insulin-like growth hormones (IGF-I and IGF-II).	Quinto et al., 2018 [196]
Polyphenols	Catechins, flavonoids, and anthocyanins	Green tea; mango; corn	0.5 g/kg for tilapia: 50 g/kg; 5 g/kg; 2 g/kg for grass carp	Plants can enhance fish species’ immune defense and antioxidant systems as a source of polyphenols. Green tea is widely used due to its high polyphenolic content. Evaluating purified polyphenols from vegetable sources is necessary to identify the components responsible for the immune and antioxidant responses in different species, aiding in the development of functional foods for aquaculture. Determining optimal doses for each species and analyzing the feed matrix’s influence on response variables is also crucial.	Lizárraga-Velázquez et al., 2018 [197]
Pigments	Astaxanthin	Crab meal, shrimp meal, oil, and seaweed.		This carotenoid helps fish’s good health and rapid growth, together with the color it provides, especially to the salmon family. It is an excellent antioxidant that potentiates its function, combined with vitamins A and E. It is mainly found bound to specific proteins.	Alanes-Oña, 2020 [198]
Pigments	Astaxanthin	Microalgae and foods that consume it, such as red trout, salmon, or crustaceans		Astaxanthin is the most potent antioxidant carotenoid for free radical scavenging: 65 times more potent than vitamin C. This compound can inhibit certain cancers and positively impact degenerative diseases. It protects membrane phospholipids and other lipids against peroxidation and contributes to terminating the induction of inflammation in biological systems. Additionally, it may have therapeutic effects against cardiovascular disease and is reported to protect against LDL cholesterol oxidation and oxidative stress.	Alanes-Oña, 2020 [198]
Fatty acids	Fatty acids n-3	PUFA from marine origin	400–3300 mg/día	In animal research studies, EPA and DHA appear to protect brain cells after stroke, especially if given very early. However, their effects as a treatment for stroke in humans have yet to be apparent.	Alvarez-Campano et al., 2020 [190]
Fatty acids	n-3 FA and n-6 FA	n-6 as part of the diet	3.9–8% of total energy intake	Consuming polyunsaturated fatty acids (PUFA) may lower blood cholesterol and reduce the risk of cardiovascular disease, but it could also lead to weight gain and inflammation. Current evidence is inconclusive, and further research is needed to understand the full health effects of increased PUFA intake.	Adbelhamid et al., 2018 [191]
Fatty acids	Fatty acids n-3	Fish oil (supplementation)		Oral omega-3 fatty acid supplementation may help prevent vascular access blockage by reducing the risk of thrombosis and stenosis.	Tam et al., 2018 [194]
Fatty acids	Fatty acids n-3	Fish oil (supplementation)		Recurrent cycles of infection and inflammation are thought to worsen lung function in patients with cystic fibrosis. Using n-3 FA and fish oil derivatives may counteract inflammation and benefit chronic inflammatory diseases, including cystic fibrosis. A 12-month study reported reduced pulmonary exacerbations and antibiotic use when taking omega-3 supplements compared to placebo.	Watson and Stackhouse, 2020 [199]
Fatty acids	Fatty acids n-3	Fish oil		Head and neck cancer can affect the oral cavity, throat, or larynx. Complications such as infections and pneumonia are common. The possibility of adding amino acids, n-3 fatty acids, and nucleotides to the diet has been analyzed to determine their potential for improving postoperative recovery. This nutritional strategy would aid recovery and reduce the number of days of hospitalization compared to a control diet.	Howes et al., 2018 [200]
Fatty acids	Fatty acids n-3 (DHA y EPA)	Obtained from fish and, in some cases, combined with antioxidants.		Ten studies involving 1015 adults with acute respiratory distress syndrome (ARDS) to compare the effects of immunonutrition with standard feeding. The studies compared standard nutrition with supplemental nutrition containing omega-3 FA or a placebo and no antioxidants. The study found uncertainty regarding the long-term survival benefits, impact on intensive care unit stay duration and ventilator dependency, and potential harm associated with this type of nutrition.	Dushianthan et al., 2019 [201]
Fatty acids	Fatty acids n-3 (DHA y EPA)	As a supplement or addition to food		Intake of n-3 during pregnancy may reduce the risk of preterm and newborns with low weight. It is essential to explore different ways of increasing n-3 intake during pregnancy.	Middleton et al., 2018 [202]
Fatty acids	Fatty acids n-3	Fish oil and fatty fish diet		A trial showed that adding fish oil (n-3 marine fatty acid) to asthmatic patients’ diets did not improve asthma symptoms.	Woods et al., 2000 [203]
Fatty acids	Linolenic acid (LA) (n-6), α-linolenic ac. (ALA) (n-3)	500 mg/day of EPA + DHA in adults, not less than 300 mg in mothers and wet-nurses, and 150 mg/day in lactating and schoolchildren.		Linolenic acid (LA) (n-6) and α-linolenic acid (ALA) (n-3) are essential fatty acids, as humans or other higher animals cannot synthesize them. In the human body, these fatty acids give rise to arachidonic acid (ARA n-6), eicosapentaenoic acid (EPA, n-3), and docosahexaenoic acid (DHA n-3). Locally acting bioactive signaling lipids called eicosanoids derived from these fatty acids also regulate various homeostatic processes. Generally, ARA gives rise to pro-inflammatory eicosanoids, while EPA and DHA give rise to anti-inflammatory eicosanoids.	Valenzuela and Samhueza, 2009; Russo, 2009; Abdelhamid, 2018; Miles, 2021 [191,204,205,206]
Fatty acids	Omega-3 fatty acids: eicosapentaenoic Acid (EPA) and Docosahexaenoic acid (DHA)	Sardine, Mackerel, Herring, Anchovy, Salmon, Sablefish, Salmon, Cod Liver, Herring Oils.	g (EPA/DHA)/100 g of raw fish: 3.3, 2.5, 1.7, 1.4, 1.4, 1.4, and 1.4. g (EPA/DHA)/100 g of oil: 44.2, 19.9, 18.5, and 11.4.	Lipoproteins have been reduced in patients with a diet rich in n-3 fatty acids. This reduction in hypertriglyceridemia is due to decreased hepatic triglyceride synthesis, increased plasma clearance, and activation of peroxisome proliferator-activated receptors (PPAR). The slight elevation of LDL with n-3 fatty acids is associated with the rapid conversion of VLDLc to LDLc, although this has only been tested in pigs and not humans. Omega-3 results in the production of smaller VLDL particles that are more susceptible to conversion to LDLc. For hypertensive patients, doses between 3 and 4 g daily of EPA/DHA have been used for periods ranging from 4 weeks to 1 year. However, in some patients, it increased the risk of stroke due to arterial hypertension. Dietary supplementation with n-3 has a hypotensive effect in hypertensive patients.	Nasif-Hadad and Meriño-Ibarra 2003 [207]
Fatty acids	n-3 FA and n-6 FA	The captive trout’s diet was commercialized in Perugia, Italy. The wild trout was caught in the Nero River, Italy.	SFA: 761.5, MUFA 433.9, PUFA 1560.6, n-3 n1234.9. n-6 157.8, w-3/w-6 7.8	The fatty acid profile in fish reflects the composition of fatty acids in their diet. Some variables indicate that the incorporation of FA into the tissue is carried out under certain metabolic effects.	Dal Bosco et al., 2013 [208]
Fatty acids	Omega-3 fatty acids: Docosahexaenoic Acid (DHA)	Fish oils (DHA)	DHA = 2000–3600 mg/d	Retinitis pigmentosa is one of several inherited eye diseases characterized by progressive degeneration of the photoreceptors located in the retina, causing severe vision loss and leading to blindness. So far, there is no treatment for this health problem. Vitamin A, fish oils, or both may help slow the progression of this group’s vision loss. Two trials evaluated the effect of DHA.	Schwartz et al., 2000 [189]
Bioactive peptides	Peptides	Fish by-products		The waste from the fishing industry contains fatty acids and proteins, which are very unstable (rancidity), so hydrolysis has been chosen to separate the fatty acids and proteins. In such a way that active peptides have been obtained as an energy source, nitrogen has physiological activity such as antioxidants, anticoagulants, antimicrobials, antidiabetics, and anticancer. These have been used to elaborate fish, poultry, and swine feeding concentrates.	Cai et al., 2015; Goosen et al., 2014; Bringas-Alvarado et al., 2018 [209,210,211]

## Data Availability

Not applicable.
